# Interplay between Host Structure, Oxygen Quenching, and Triplet–Triplet Annihilation Upconversion in Hybrid Polymer Hosts

**DOI:** 10.1021/acsaom.6c00025

**Published:** 2026-04-08

**Authors:** Georgina H. Burgoyne Morris, Larissa Gomes Franca, Abigail R. Collins, Rachel C. Evans

**Affiliations:** Department of Materials Science & Metallurgy, 2152 University of Cambridge, 27 Charles Babbage Road, Cambridge CB3 0FS, U.K.

**Keywords:** triplet−triplet annihilation upconversion, oxygen quenching, organic−inorganic hybrid polymers, phosphorescence, ureasil

## Abstract

The ability to convert low-energy photons to those of higher energy through triplet–triplet annihilation upconversion (TTA-UC) is of significant interest for diverse applications including solar energy harvesting, sensing, and anticounterfeiting. However, the efficiency of TTA-UC under ambient conditions is often severely limited due to the quenching of excited triplet states by molecular oxygen. Therefore, when designing TTA-UC systems, it is crucial to effectively characterize the extent of oxygen quenching and to use these insights to drive material design that effectively prevents the permeation of oxygen. In this work, we investigate the oxygen barrier properties of three organic–inorganic hybrid polymers known as ureasils, previously determined to be effective TTA-UC hosts under ambient conditions. Through both direct oxygen permeation measurements and kinetic analysis of the phosphorescence quenching of palladium­(II) octaethylporphyrin (PdOEP), we investigate how the ureasil structure, particularly with respect to silica content and molecular weight and branching of the polymer chains, affects the bulk and local oxygen permeabilities. We also demonstrate the interplay of oxygen quenching with the wider TTA-UC process, confirming that the variation in oxygen permeability is the primary factor affecting ambient TTA-UC efficiency between different ureasil structures. This emphasizes the importance of considering oxygen barrier properties as a key metric in the design of future host materials for more efficient ambient TTA-UC systems.

## Graphical abstract

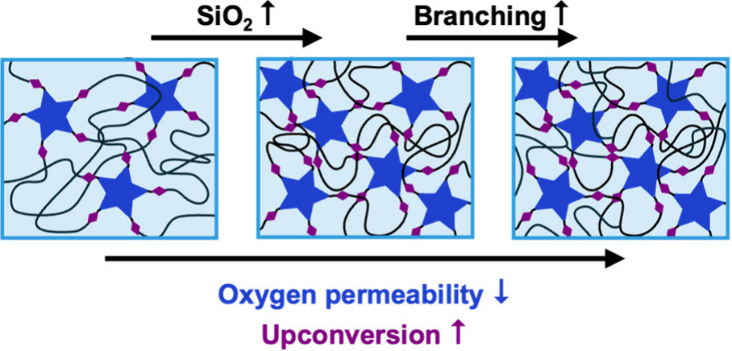

## Introduction

Triplet–triplet annihilation (TTA-UC) is a process by which two low-energy photons can be converted to a single photon of higher energy^1,2^ and has potential uses in many applications, including photovoltaics,^3,4^ photocatalysis,^5,6^ phototransistors,^7^ optical computing,^8^ anticounterfeiting,^9^ sensing,^10^ bioimaging^11^ and drug delivery.^12^ As shown in Figure 1, the TTA-UC process relies on multiple energy transfer steps between a triplet sensitizer, such as palladium­(II) octaethylporphyrin (PdOEP), and a suitably matched emitter, such as diphenylanthracene (DPA). This typically requires rapid molecular collisions and therefore occurs efficiently in degassed liquid solutions, with upconversion quantum yields (Φ_UC_) reaching ∼40% (of a maximum 50%).^13–15^ However, liquid systems lack mechanical robustness and pose risks of leakage or evaporation, introducing challenges for practical applications.^16^ These weaknesses can be overcome by immobilizing the chromophores in a solid-state host. Suitable hosts should be transparent, mechanically stable, and capable of dispersing dissolved chromophores without aggregation. Crucially, they should also enable high rates of either molecular or triplet exciton diffusion, to allow intramolecular triplet–triplet energy transfer (TTET) and triplet–triplet annihilation (TTA) to occur efficiently.^17^ Typical hosts are based on organic polymers but vary significantly in form, ranging from flexible elastomers,^18,19^ to rigid resins or glasses,^20,21^ to solvated or dry gels.^22,23^

**Figure 1:**
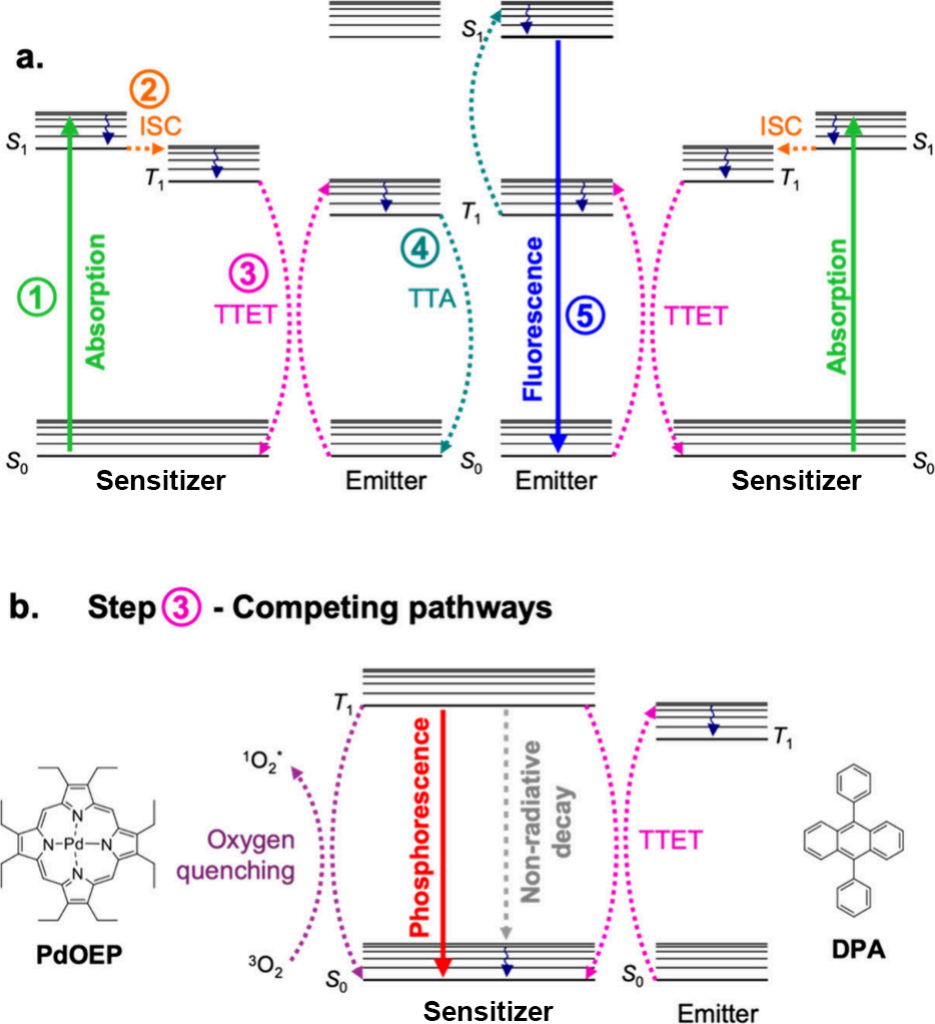
(a) Jablonski diagram for the TTA-UC process. Absorption of an incident photon by the sensitizer (step 1, green solid arrow) results in the population of the singlet excited state (*S*_1_), which undergoes rapid intersystem crossing (ISC, step 2, orange dashed arrow) to the first triplet excited state (*T*_1_). Triplet–triplet energy transfer (TTET, step 3, pink dashed arrows) then occurs from the sensitizer to an emitter with suitably matched triplet energy levels. Two triplet-excited emitters may undergo triplet–triplet annihilation (TTA, step 4, teal dashed arrows) such that one emitter relaxes to its ground state and the second emitter is excited to its *S*_1_ state, before relaxing via the emission of a photon (step 5, blue solid arrow). (b) Several other relaxation processes can compete with TTA-UC, including radiative relaxation of the *T*_1_ state via phosphorescence (red solid arrow), nonradiative relaxation (gray dashed arrow), or quenching by molecular oxygen (purple dashed arrows), instead of TTET to the emitter. Solid arrows represent radiative processes, dashed arrows represent nonradiative processes, and wavy arrows represent vibrational relaxation.

Another important factor that must be considered when working in air is the presence of molecular oxygen, which will quench long-lived photoexcited species, particularly triplet excited states (as shown for the sensitizer in Figure 1b), causing significant reductions in TTA-UC efficiency.^24^ It is therefore crucial to understand and minimize the impact of oxygen quenching on any TTA-UC system designed to operate under ambient conditions. Different approaches have been used to inhibit the detrimental effect of O_2_ quenching on the TTA-UC efficiency, including encapsulation of the upconverting system in an oxygen barrier material such as poly­(vinyl alcohol) (or derivatives thereof)^8,25^ or glass,^5^ often following air-free fabrication,^18^ or inclusion of oxygen scavengers.^26,27^ However, from a practical standpoint, it is much more desirable for the host material itself to act as a barrier to prevent oxygen quenching. In such systems, the host’s ability to exclude oxygen is often evaluated by comparing upconversion performance in air with that under air-free conditions,^28^ or by comparing the sensitizer’s phosphorescence lifetime in the host of interest with literature values for deoxygenated polymer hosts.^29,30^ However, detailed quantitative analysis of oxygen permeability, either directly on the bulk scale or indirectly in the local environment of the chromophores, is not typically performed.

Heavy transition metal complexes such as platinum­(II) or palladium­(II) metalloporphyrins are commonly used as sensitizers for TTA-UC due to their high quantum yields of intersystem crossing.^16,31^ Oxygen quenching of metalloporphyrin triplets is well-studied and has been characterized in films of organic polymers such as poly­(vinyl chloride),^32–36^ poly­(methacrylate)­s,^35,37^ poly­(styrene)^32,35^ and cellulose derivatives,^33,34,36^ as well as inorganic and hybrid materials such as sol–gel derived silica,^38^ polyphosphazenes^39^ and silicones.^32,35^ The oxygen sensing properties of metalloporphyrins in these matrices have found numerous applications including biosensors,^40,41^ food packaging,^42,43^ and pressure-sensitive paints,^44–46^ as well as in the characterization of oxygen barrier properties of polymeric and composite films.^47,48^ From detailed analysis of the quenching kinetics using both time-resolved and steady-state phosphorescence measurements, it has been found that many hosts present a heterogeneous distribution of chromophore sites, attributed to variations in the local solubility or diffusivity of oxygen.^33,34^

In this study, we examine the relationship between the host structure and TTA-UC efficiency and attempt to correlate this with the local oxygen environment within the host matrix. We use a family of organic–inorganic hybrid polymers, known as *ureasils*, as the host, which have previously been investigated for optical applications including luminescent solar concentrators,^49,50^ bioimaging,^11^ and visible light communications.^51,52^ Ureasils generally consist of nanoscale siliceous domains cross-linked via urea bonds by poly­(oxyalkylene) chains. This hybrid nature provides compromise between the flexibility and mobility of organic polymers and the improved barrier properties of inorganic materials. It is also likely to introduce an inherent inhomogeneity in chromophore sites, with properties dependent on the chromophores’ proximity to the siliceous nanodomains. We recently demonstrated that ureasils with organic poly­(propylene glycol) segments of molecular weight (MW) in the range 2000–5000 g mol^–1^ are effective TTA-UC hosts under ambient conditions.^53^ These host materials exhibited high optical transparency (∼90% transmittance from 300 to 800 nm for ∼1 mm thick samples), making them suitable for TTA-UC across the visible and near-infrared regions, and achieved a maximum Φ_UC_ of 1.86 ± 0.05% at an excitation power of 1 W cm^–2^ using the PdOEP/DPA chromophore (0.1 mM/10 mM) pair.^53^ Notably, we observed that higher TTA-UC efficiencies were obtained for ureasils with lower-MW organic chains and consequently higher silica contents. In other hybrid polymers, a direct correlation between increased silica content and decreased oxygen permeability has been reported.^54^ Herein, we use a combination of direct measurement of bulk oxygen permeability, combined with time-resolved emission lifetime analysis to probe the local oxygen environment around the sensitizer to determine if there is a direct relationship between the bulk ureasil structure, the chromophore environment, and TTA-UC efficiency. With this work, we aim to improve the understanding of the competing factors affecting the TTA-UC process in ureasil hosts, aiding the design of future host materials.

## Results and Discussion

### Direct Measurement of Bulk Oxygen Permeability: Undoped Ureasils

The ureasil host structures investigated in this study are listed in Figure 2. The synthesis and structural properties of ureasils have previously been investigated in detail.^55,56^ On condensation of the siloxane end-groups of a polymeric sol–gel precursor, amorphous siliceous nanodomains form. From X-ray diffraction studies of these materials, the local ordering of the silica structural units has been found to typically persist over a coherence length on the order of ∼10–20 Å,^55,56^ with the specific ureasils investigated in this study showing coherence lengths of ∼16–17 Å.^53^ The siliceous domains are therefore likely to be too small for significant Rayleigh scattering, which affords them with a high optical transparency. DU(2000) and DU(4000) both consist of linear poly­(propylene glycol) chains, but of different molecular weights, leading to different silica contents, while TU(3000) has the same silica content as DU(2000) but a tribranched polymer structure. As such, a comparison of these three hosts allows the effects of silica content and branching to be probed independently.

**Figure 2:**
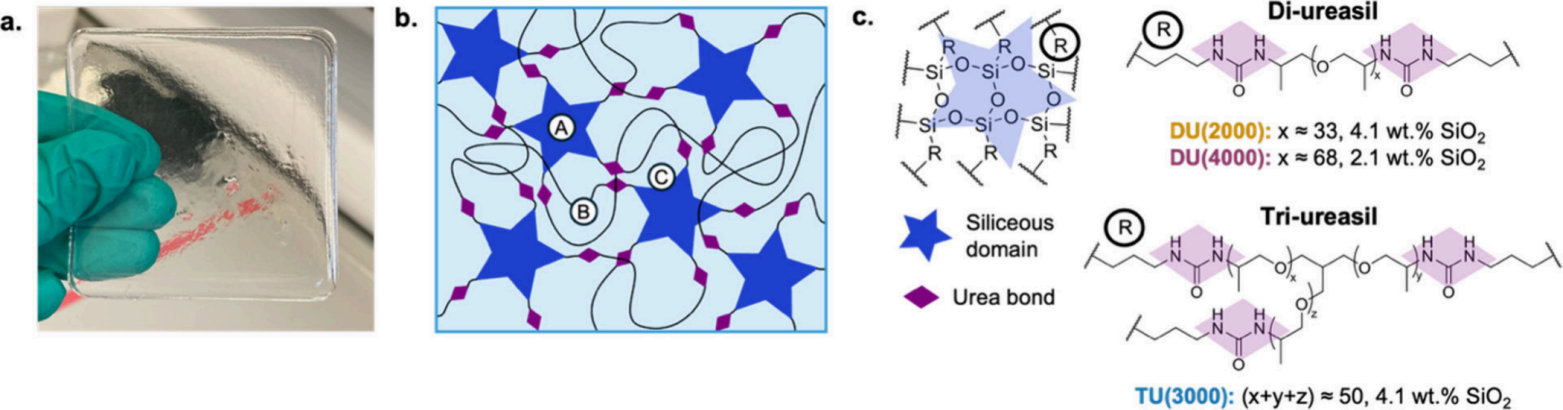
Structure of ureasil TTA-UC hosts. (a) Photograph of a typical ureasil sample. (b) Schematic representation of the ureasil structure. Siliceous nanodomains are linked via urea bonds by poly­(propylene glycol) chains, giving rise to multiple types of sites that may be potentially occupied by dissolved chromophores: (A) inside siliceous domains, (B) in organic regions, or (C) at the boundary between the two. (c) Chemical structures of ureasils investigated in this work, with the corresponding silica contents.

The inherent oxygen barrier properties of the undoped ureasil host materials on a bulk scale were measured first. For free-standing, monolithic films, such as the ureasils investigated here (see Figure 2a), the oxygen permeability can be obtained by direct measurement of the oxygen transmission rate (OTR) through the film, from a region of high oxygen partial pressure (*p*_O_2__) to one of low *p*_O_2__. The steady-state OTR depends on the partial pressure change (Δ*p*_O_2__) and film thickness (*t*), as well as the inherent materials properties. The latter are represented by the oxygen permeability coefficient (*P*_O_2__), which, for a homogeneous film, can be calculated from^57^

1 $$P_{O_{2}}=\frac{\mathrm{OTR}}{{\Delta p}_{O_{2}}}\times t$$

From the calculated *P*_O_2__ values (see Table S1 in the Supporting Information (SI) for details), two key trends could be observed. First, comparing DU(4000) (2.1 wt % SiO_2_) and DU(2000) (4.1 wt % SiO_2_), it can be seen that the increase in silica content results in a substantial decrease in *P*_O_2__ from 26.6 ± 0.7 barrer in DU(4000) to 13.5 ± 0.5 barrer in DU(2000), *i.e*. increased silica content improves oxygen barrier properties. The *P*_O_2__ is also slightly lower, at 11.5 ± 0.3 barrer, in TU(3000) than in DU(2000), despite them having equal silica contents, suggesting branching also decreases oxygen permeability. This is potentially a result of the decreased length of free poly­(propylene glycol) chains in the tribranched structure, which has been found to lead to a higher glass transition temperature (*T*_g_), and elastic and bending moduli,^53^ and thus likely also reduces oxygen mobility. For all ureasils, the *P*_O_2__ values are comparable to organic rubbery polymers commonly used as TTA-UC hosts, *e.g*. poly­(ether)-based poly­(urethanes) (∼3–30 barrer, depending on hard segment structure, for a soft segment MW of 2000),^58^ poly­(alkyl acrylates) (∼1–80 barrer),^59^ and poly­(caprolactone) (∼25 barrer),^59^ though it is worth noting that these values were not obtained in the specific context of TTA-UC host characterization, as such measurements are not common practice. These results suggest that, as has been proposed for composites of polymers with silica nanoparticles,^48^ the permeation pathway of oxygen through ureasils is primarily through the organic regions, with the siliceous domains acting as “obstacles”, increasing the tortuosity of the oxygen molecules’ path through the film, and thus decreasing the permeation rate on a bulk scale.

### Oxygen Quenching of Sensitizer Phosphorescence: PdOEP-Doped Ureasils

The effects of oxygen on the time-resolved phosphorescence of ureasils doped with 0.1 mM PdOEP, initially in the absence of any TTA-UC emitter, were next investigated. To do this, phosphorescence decay curves were measured for samples which, having been fabricated in air, were cut to size and placed inside a cuvette, which was then sealed and deaerated by purging with a constant flow of N_2_ for 4 h. These decays were compared to those measured directly in air. Figure 3 shows representative resulting phosphorescence decay curves and averages of fitted lifetime components, while all measured decays are given in Figures S1 and S2, and the multiexponential fitting parameters for these decays are given in Tables S2 and S3 (see SI). Under N_2_, in all ureasil samples the dominant phosphorescence lifetime was 1.1 – 1.5 ms (fractional contribution, *f* = 93–98%), consistent with previous reports for PdOEP in various polymer hosts in the absence of oxygen.^33^ This lifetime was therefore taken to be the unquenched phosphorescence lifetime and was designated τ_0_. In all ureasils, some repeats showed an additional shorter lifetime of 0.5–0.8 ms (*f* = 2–7%). This was attributed to local quenching from small amounts of residual oxygen and as such was designated τ_res_. To investigate whether other samples, which showed monoexponential decays in the absence of oxygen, might show a similar deviation in the presence of small amounts of oxygen, the phosphorescence decays in TU(3000) were measured as a function of time upon reexposure to air (see Figure S3 and Table S4, Supporting Information). On initial exposure (0 min), the long-lived decay was largely retained, but deviated slightly from monoexponential behavior, supporting the assignment of the similar decay in DU(2000). These results suggest that, in the absence of oxygen, there is no significant inhomogeneity in the chromophore sites. However, since the siliceous domains in ureasils would be expected to provide very different chromophore environments to the organic regions, the observed homogeneity suggests that the chromophores are in fact confined to just one of these regions. As the organic material contributes a much larger fraction of the overall ureasil mass, it is most likely that this is where the chromophores are localized.

**Figure 3:**
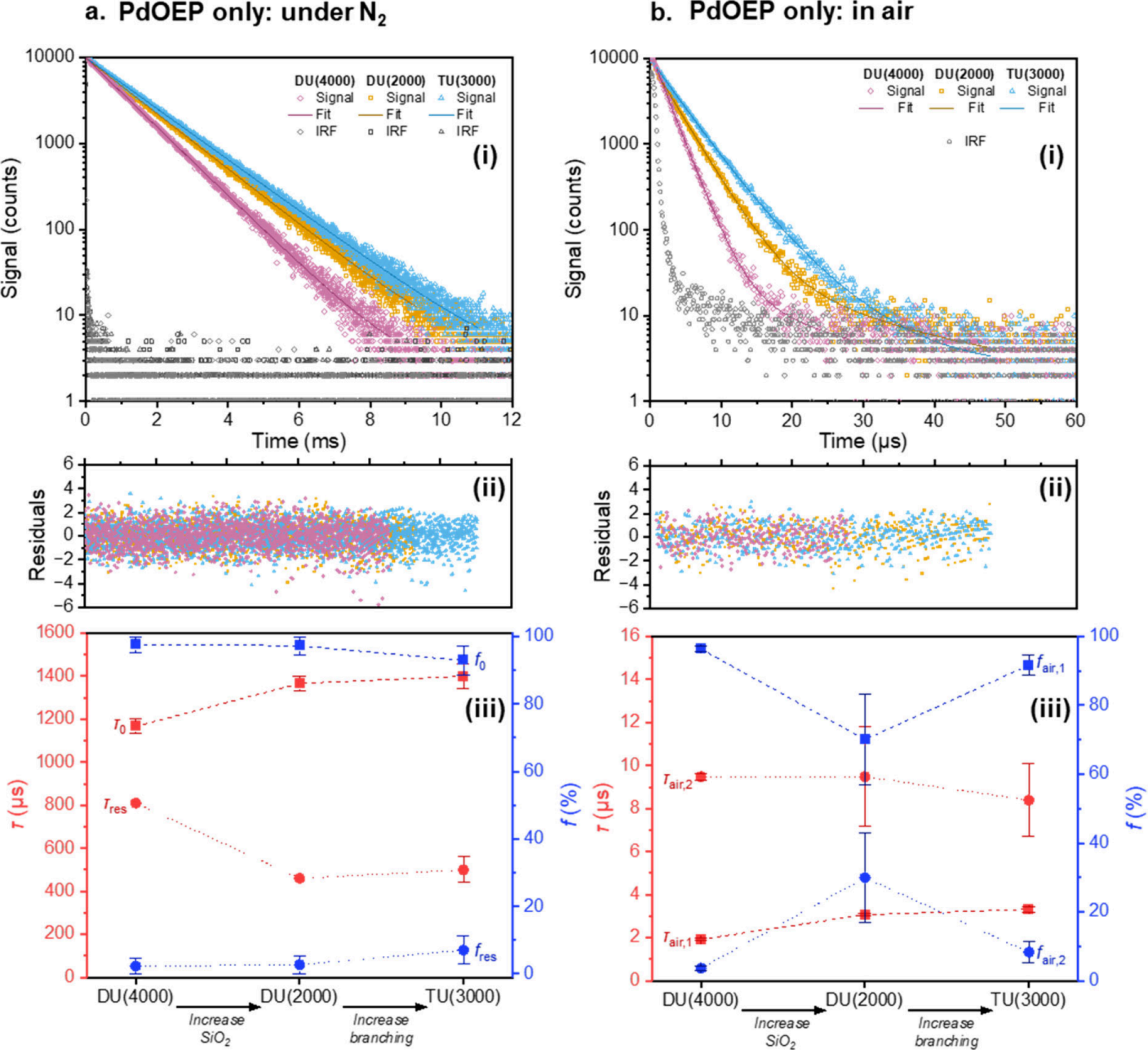
Influence of atmospheric oxygen on the phosphorescence decay kinetics of PdOEP (0.1 mM) in ureasil hosts measured in a sealed cuvette after (a) purging with N_2_ for 4 h and (b) in air: (i) Phosphorescence decays and Instrument Response Function (IRF) (open symbols) and corresponding exponential fits (solid lines), λ_ex_ = 532 nm, λ_em_ = 665 nm; (ii) corresponding residuals for decay fits; (iii) extracted lifetime components (red symbols) and corresponding fractional contributions (blue symbols). No errors are given in τ_res_ for DU(2000) or DU(4000), as this lifetime component was only evident in a single repeat for each host, and thus, no meaningful error could be generated.

In air, the major contribution (*f* = 70–96%) to all decays was from a short lifetime (τ_air,1_) between 2 and 4 μs, which we assign to oxygen-quenched triplet states in the most permeable local environments. Following the observed trends in bulk oxygen permeability, τ_air,1_ showed a significant increase with increasing silica content from 1.92 ± 0.05 μs in DU(4000), to 3.07 ± 0.06 μs in DU(2000), and also increased with branching to 3.32 ± 0.13 μs in TU(3000), suggesting a decrease in oxygen quenching in both cases. To quantify this further, the oxygen quenching rate constant, *k*_O_2__, was calculated:

2 $$k_{O_{2}}=\frac{1}{\tau_{\mathrm{air},1}}-\frac{1}{\tau_{0}}$$

The resulting values were 5.19 ± 0.13 × 10^5^ s^–1^ in DU(4000), 3.26 ± 0.06 × 10^5^ s^–1^ in DU(2000), and 3.00 ± 0.12 × 10^5^ s^–1^ in TU(3000), supporting the assertion that the rate of oxygen quenching decreases substantially with increased silica content and slightly with increased branching.

All hosts also showed contributions from a second, longer lifetime (τ_air,2_ = 8.4–9.5 μs, *f* = 4–30%), suggesting that there is a variation in the rate of oxygen quenching across different chromophore sites in each host. For example, as discussed above, oxygen may primarily diffuse through the more permeable organic regions, meaning chromophores in sites closer to siliceous domains (e.g., site C in Figure 2b) may experience a lower local oxygen concentration, reducing the quenching rate.

The connection between host oxygen permeability and phosphorescence quenching, especially of metalloporphyrins, has been thoroughly investigated in the literature,^33,34,47,48^ and various models exist to analyze the data. The simplest of these, under the assumption of dynamic, diffusion-controlled quenching, is the Stern–Volmer equation.^60^ In the context of a system in equilibrium with gaseous oxygen of low to moderate partial pressure, this can be written in terms of partial pressure and oxygen permeability coefficient (see SI, section 2 for full derivation):^33^

3 $$\frac{I_{0}}{I}=\frac{\tau_{0}}{\tau }=1+K_{\mathrm{SV}}^{\mathrm{gas}}p_{O_{2}}$$

4 $$K_{\mathrm{SV}}^{\mathrm{gas}}=\tau_{0}f_{Q}N_{A}4\pi \left(r_{L}+r_{O_{2}}\right)P_{O_{2}}$$

where *I* and *I*_0_ are the emission intensities in the presence and absence, respectively, of oxygen at partial pressure *p*_O_2__, and τ and τ_0_ are the equivalent lifetimes. *f*_*Q*_ is the quenching efficiency factor, *N*_A_ is Avogadro’s number, *r*_L_ and *r*_O_2__ are the molecular radii of the luminophore and oxygen, respectively, and *P*_O_2__ is the oxygen permeability coefficient (as in eq 1). Alternatively, for metalloporphyrins in several organic polymer hosts, including poly­(vinyl chloride) and cellulose derivatives, the Freundlich isotherm has been found to give a better fit:^33^

5 $$\frac{I_{0}}{I}=1+{\left(K_{F}^{\mathrm{gas}}p_{O_{2}}\right)}^{\beta }$$

6 $$K_{F}^{\mathrm{gas}}=\tau_{0}{fN}_{A}4\pi \left(r_{L}+r_{O_{2}}\right)P_{O_{2}}p_{O_{2}}$$

where β < 1 acts as a measure of inhomogeneity, accounting for the heterogeneous distribution of sites observed in these host materials. Crucially, provided other parameters are known or can be estimated, both analyses provide a route to predicting *P*_O_2__ from analysis of lifetime data at different oxygen partial pressures. In the literature, this has typically involved fitting of multiple data points as a function of partial pressure, requiring a mechanism for the control and measurement of oxygen partial pressure.^47^ Here, however, for the sake of simplicity, we only compare the phosphorescence in air (*p*_O_2__ = 0.21 atm) with that under N_2_ (*p*_O_2__ ≈ 0). For each host, the fitted emission decay curves under air and N_2_ were integrated to obtain intensities *I = I*_air_ and *I*_0_ respectively, and the corresponding *P*_O_2__ values were calculated using eq 3–4 (henceforth referred to as “Stern–Volmer Intensity Analysis”) or 5–6 (“Freundlich Intensity Analysis”). For the Freundlich Intensity Analysis, a value of β = 0.95 was chosen, based on previous fitting of this equation for PdOEP and PtOEP in various organic polymer hosts.^33^ Similarly, the following values from the literature were assumed: *f*_Q_ = 1, *r*_PdOEP_ = 350 pm,^32,61^ and *r*_O_2__= 200 pm.^62^ In addition, to investigate the local oxygen permeability in the majority environment of the chromophores, Stern–Volmer Lifetime Analysis was also applied to individual decay components, taking τ to be τ_air,1_, representing the highest degree of quenching, and using τ_0_ as assigned to the decay fits under N_2_. The corresponding results for each of these three analyses for the ureasil hosts are shown in Figure 4 and Table S5 (see SI), alongside the bulk *P*_O_2__ results from direct oxygen permeation measurements. This allows for both the evaluation of the effectiveness of these photophysical analyses in predicting trends in bulk oxygen barrier properties, and comparison of the bulk values with those experienced locally at the chromophore sites.

**Figure 4:**
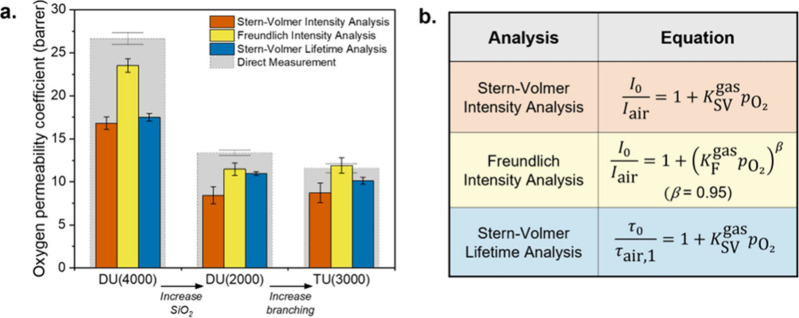
Kinetic analysis of the phosphorescence decays of PdOEP in ureasil hosts in N_2_ and air. (a) Oxygen permeability coefficients determined from (b) three different analytical methods. Results of bulk oxygen permeation analysis (gray bars) are shown for comparison.

The trend in oxygen permeabilities between the three ureasil hosts is similar regardless of method, with the *P*_O_2__ decreasing substantially with increasing silica content (from DU(4000) to DU(2000)).While the two intensity-based analyses do show a slight increase in *P*_O_2__ from DU(2000) and TU(3000), contrary to the direct measurements and Stern–Volmer Lifetime Analysis, the variation is negligible relative to the margins of error, so the values across the two hosts can be viewed as comparable. All three photophysical methods therefore accurately reflect both the relative trends and orders of magnitude of the bulk oxygen permeabilities. Of the different methods, the closest agreement with the bulk values was seen for the Freundlich Intensity Analysis. This reflects the inhomogeneity in chromophore sites present in the ureasil hosts, which is accounted for in this model. In fact, with the exception of TU(3000), all values from this analysis still fell below the bulk values, suggesting that the inhomogeneity in these hosts may in fact be greater than that accounted for through the assumed β value, which is reasonable given their hybrid nature. However, without further measurements at multiple known oxygen partial pressures, which are not accessible in our experimental setup, it is not possible to accurately fit the true value of β. Furthermore, it may simply be the case that the bulk oxygen permeabilities are larger than those experienced in the local environment of the chromophores.

With respect to the local oxygen permeabilities experienced by the chromophores, the most insight can be gained from the Stern–Volmer Lifetime Analysis. As this method considers only the majority chromophore environments, it does not rely on assumptions surrounding the degree of inhomogeneity in the host. Furthermore, while the overall decay intensity under N_2_ may be affected by minor contributions from residual oxygen quenching, leading to an underprediction of *P*_O_2__, the Stern–Volmer Lifetime Analysis just uses the longest, unquenched lifetime component, making it less sensitive to incomplete deoxygenation. Interestingly, the oxygen permeabilities obtained using the Stern–Volmer Lifetime Analysis fall below the bulk values for all hosts, despite the fact they reflect the most extreme quenching conditions and so may be expected to overpredict. This suggests that the chromophores may indeed experience some local shielding from oxygen, perhaps by the silica nanodomains, reducing the local oxygen permeability. These results clearly demonstrate, regardless of the analytical method used, that the structure of the ureasil host plays an important role in determining the oxygen permeability, not just on a bulk scale but also in the local environments of the chromophores, which has direct implications for oxygen quenching of the sensitizer triplet state.

### Interplay of Oxygen Quenching and TTET: TTA-UC Pair-Doped (S+E) Ureasils

In the full TTA-UC system, the effect of the emitter and the interplay between oxygen quenching and TTET, must also be considered. To investigate this, the phosphorescence lifetimes were measured for the three ureasil hosts codoped with PdOEP (0.1 mM) and DPA (10 mM), in air and under N_2_. Representative decays and average lifetime components are shown in Figure 5, with all decays and fitting parameters detailed respectively in Figures S4–5 and Tables S6–7 (see SI).

**Figure 5:**
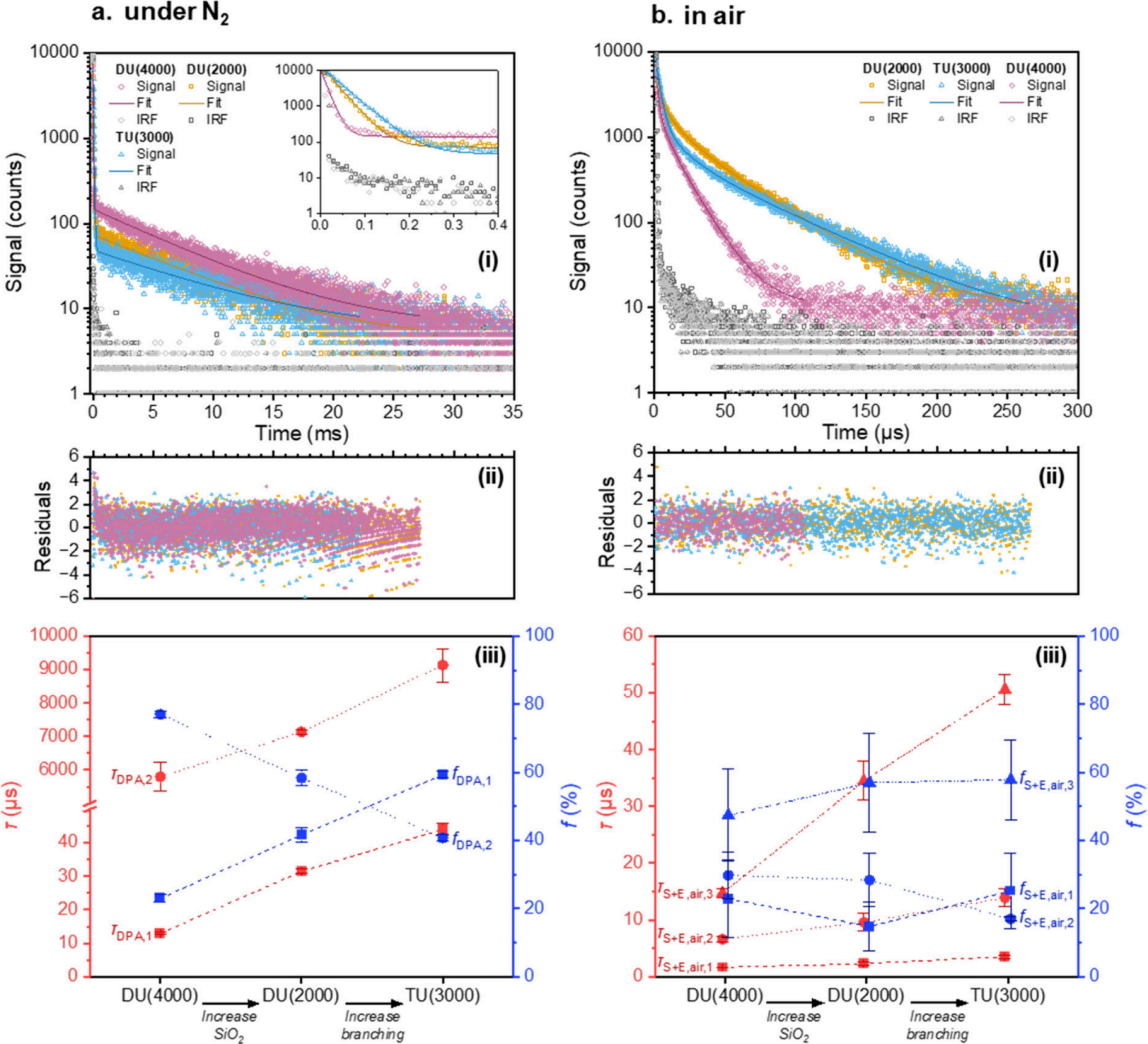
Phosphorescence lifetime analysis of PdOEP (0.1 mM) in the presence of DPA in ureasil hosts measured in a sealed cuvette after (a) purging with N_2_ and (b) in air. (i) Phosphorescence decays and IRF (open symbols) and corresponding exponential fits (solid lines), λ_ex_ = 532 nm, λ_em_ = 665 nm; (ii) corresponding residuals for decay fits; (iii) extracted lifetime components (red symbols) and corresponding fractional contributions (blue symbols).

Under nitrogen, the phosphorescence decays for all three hosts showed two clear regimes: a short lifetime component (τ_S+E,1_) on the order of tens of μs, and a longer component (τ_S+E,2_) of multiple ms. τ_S+E,1_ (13–44 μs, *f* = 23–59%) can be attributed to dynamic quenching of the PdOEP triplet states due to triplet–triplet energy transfer to DPA, as would be expected.^63^ As for oxygen quenching, the rate constant for TTET can therefore be calculated:

7 $$k_{\mathrm{TTET}}=\frac{1}{\tau_{S+E,1}}-\frac{1}{\tau_{0}}$$

This gives *k*_TTET_ as 7.66 ± 0.21 × 10^4^ s^–1^ in DU(4000), 3.11 ± 0.07 × 10^4^ s^–1^ in DU(2000), and 2.20 ± 0.09 × 10^4^ s^–1^ in TU(3000), reflecting the trend seen in *k*_O_2__. The comparison of quenched and unquenched lifetimes can also provide insight into the diffusion properties of the host. In this case, under the assumption of bimolecular diffusion-controlled quenching, the Stern–Volmer^60^ and Smulochowski^64^ equations can be combined to give:

8 $$\frac{\tau_{0}}{\tau_{S+E,1}}=1+\tau_{0}f_{Q}N_{A}4\pi rD_{\mathrm{tot}}\left[\mathrm{DPA}\right]$$

where τ_0_, *f*_Q_, and *N*_A_ are as previously defined. *f*_Q_ was again assumed to be 1, while the collision radius *r* was taken to be 9.8 Å, the Dexter energy transfer radius previously calculated for TTET from PtOEP to DPA.^63,65^ This allowed *D*_tot_, the sum of the respective diffusivities of PdOEP and DPA, to be calculated as 10.3 ± 0.3 × 10^–9^ cm^2^ s^–1^ in DU(4000), 4.10 ± 0.09 × 10^–9^ cm^2^ s^–1^ in DU(2000), and 2.97 ± 0.12 × 10^–9^ cm^2^ s^–1^ in TU(3000). As with oxygen permeability, this shows that chromophore mobility decreases substantially with increased silica content and slightly with increased branching. Again, this can be explained by the decrease in length of the free polymer chains, leading to a reduction in mobility, as is reflected by the increase in *T*_g_ and elastic and bending moduli previously measured across this series of materials.^53^

The presence of τ_S+E,2_ (5.8–9.1 ms, *f* = 41–77%) was somewhat surprising, as, for each host, it was longer than the unquenched lifetime τ_0_ (1.1–1.5 ms, see Figure 3a). τ_S+E,2_ was also longer than the average lifetime of the upconverted DPA emission (<τ_UC_> = 3.8–6.6 ms, see Figure S7 and Table S9, Supporting Information) in each respective host, suggesting this arose from re-excitation of PdOEP after the TTET and TTA steps, due to either parasitic Förster resonance energy back-transfer from the singlet-excited DPA,^16,66^ or reabsorption of the DPA fluorescence.^67^ As shown in Figure 5a­(iii), τ_S+E,2_ becomes longer as the host mobility decreases (be that due to increased silica content or branching), which is to be expected as both the TTET and TTA steps prior to re-excitation of the PdOEP would occur at slower rates. However, despite the increase in lifetime with decreasing mobility, the fractional contribution *f*_S+E,2_ shows a clear decrease, implying less re-excitation occurs in the less mobile hosts, though the reasons behind this are still unclear. The result of this behavior is that while the TTET rate constant varies with host mobility, the overall efficiency of TTET is approximately constant (∼94–95%) across all three hosts (see Table S10, Supporting Information).

In air, the situation is more complex due to competition between oxygen quenching and TTET to DPA. As discussed above, the rate of oxygen quenching of PdOEP phosphorescence clearly varies across chromophore sites in the ureasil hosts, even in the absence of DPA, so its introduction produces yet another variable. This is reflected in the multiple exponential components used to fit the phosphorescence decays. In all hosts, three exponential lifetime components were required to fit the decays: a short component (τ_S+E,air,1_; *f* = 17–31%) of ∼2–3 μs, a slightly longer component (τ_S+E,air,2_; *f* = 17–30%) of 7–14 μs, and a third longer component (τ_S+E,air,3_; *f* = 47–57%) of 15–44 μs. It should also be noted that, while the lifetimes were fairly consistent between repeat measurements, the fractional contributions show significant variation between repeats (Figure S5 and Table S7, Supporting Information), as is reflected in the substantial errors evident in Figure 5b­(iii). In each host, the similarity between τ_S+E,air,1_ and τ_air,1_ (measured for the PdOEP-doped samples in air, see Figure 3b), suggests the dominant quenching pathway at certain PdOEP sites may be from oxygen quenching, even in the presence of DPA. τ_S+E,air,3_, meanwhile, lies much closer to τ_S+E,1_, suggesting that the corresponding PdOEP sites are primarily quenched by TTET to DPA and not by oxygen. The intermediate τ_S+E,air,3_ is more difficult to confidently assign but possibly arises from a combination of the two quenching mechanisms. To investigate whether this inhomogeneity was due to different dominant mechanisms operating in different local environments or a result of a change in behavior over the course of the decay acquisition, a series of sequential phosphorescence decays were measured for TU(3000) (see Figure S6 and Table S8, Supporting Information). A general increase in the overall lifetime could be seen over time, with the longest lifetime (τ_3_) increasingly dominating for later scans. This suggests that, with increasing irradiation time, a transition occurs from primarily oxygen-quenched behavior to dominance by TTET. This may seem counterintuitive, as the calculated quenching rate constants show that oxygen quenching is an order of magnitude faster than TTET. However, a possible explanation is that DPA is known to react with singlet oxygen to form endoperoxides, thus reducing triplet oxygen quenching over time and allowing TTET to dominate.^68–70^ The significant variation in fractional contributions between repeat measurements could therefore be explained by differences in the rate of photoconsumption of oxygen between samples, with a higher *f*_S+E,air,3_ and a lower *f*_S+E,air,1_ suggestive of more rapid oxygen scavenging in that sample.

However, these assignments of lifetime components should only be taken as tentative suggestions, particularly since there are issues with reproducibility. The substantial difference between the decays in Figure S6 (SI), despite being measured sequentially on the same sample, illustrates how even acquisition time can affect the measured decay. Previous measurements for the phosphorescence lifetimes of seemingly identical samples also gave average lifetimes of ∼60–300 μs, significantly higher than those obtained here.^53^ Regardless, it is clear that competition with oxygen quenching makes quantitative analysis of TTET to DPA difficult for measurements in air but that the interplay between TTET and oxygen leads to complicated phosphorescence behavior.

### Effects of Oxygen on Upconversion Efficiency: TTA-UC Pair-Doped Ureasils

Given the clear competition between TTET and oxygen quenching for the TTA-UC pair-doped samples, it was of interest to see how this impacted the overall upconversion efficiency. For simplicity, only the most and least mobile hosts, DU(4000) and TU(3000), respectively, were investigated, again doped with PdOEP (0.1 mM) and DPA (10 mM). Of the hosts investigated in this study, these two represent extremes in silica content, oxygen permeability and chromophore diffusivity, as well as *T*_g_ and elastic moduli.^53^ These factors are likely to affect multiple steps of the TTA-UC process. This study has already demonstrated the complex competition between oxygen quenching and TTET, both of which are promoted in more flexible, less siliceous and lower- *T*_g_ hosts, and similar competition could be expected for the TTA step, hence the importance of viewing the TTA-UC efficiency as a whole.

The upconversion emission was measured for both samples after purging with N_2_ for up to 17 h. Figure 6a shows the integrated UC emission intensity, normalized to that prior to deaeration, as a function of the deaeration time. It should be noted that the large errors apparent in the relative intensities after deaeration arise from the statistical propagation of the errors in the measured absolute intensities both before and after deaeration. As expected, both samples showed an increase in emission intensity with deaeration due to decreased oxygen quenching, with a more pronounced effect on the more permeable DU­(4000). However, for both samples, the effect of deaeration on UC emission was significantly smaller than on the sensitizer phosphorescence in the absence of emitter. This supports the idea that DPA scavenges oxygen when irradiated, diminishing the effects of oxygen quenching. As these measurements were carried out under continuous laser irradiation (1 W cm^–2^), this photoconsumption would be expected to occur much more rapidly than under the pulsed excitation conditions used for the decay measurements, further reducing the impact of oxygen quenching. To estimate the upconversion quantum yields (Φ_UC_) without the effect of oxygen quenching, Φ_UC_ values were measured in air and then multiplied by the relative intensities on deaeration. As shown in Figure 6b, while TU(3000) shows a substantially higher Φ_UC_ than DU(4000) in air, with deaeration the values for each host become closer, lying well within error of each other after 17 h’ deaeration. Although it may have been expected that DU(4000) would show a higher Φ_UC_ in the absence of oxygen, since its higher mobility would facilitate greater energy transfer efficiencies, this does not occur. Likely this offset is due to the increase in vibrational relaxation and parasitic reabsorption or back-transfer observed from the kinetic data above (Figure 5a), as is reflected in the calculated TTET efficiencies (Table S10, Supporting Information). It can therefore be concluded that the primary factor causing the difference in ambient Φ_UC_ across ureasil hosts is the variation in oxygen permeability that arises from the different ureasil structures.

**Figure 6:**
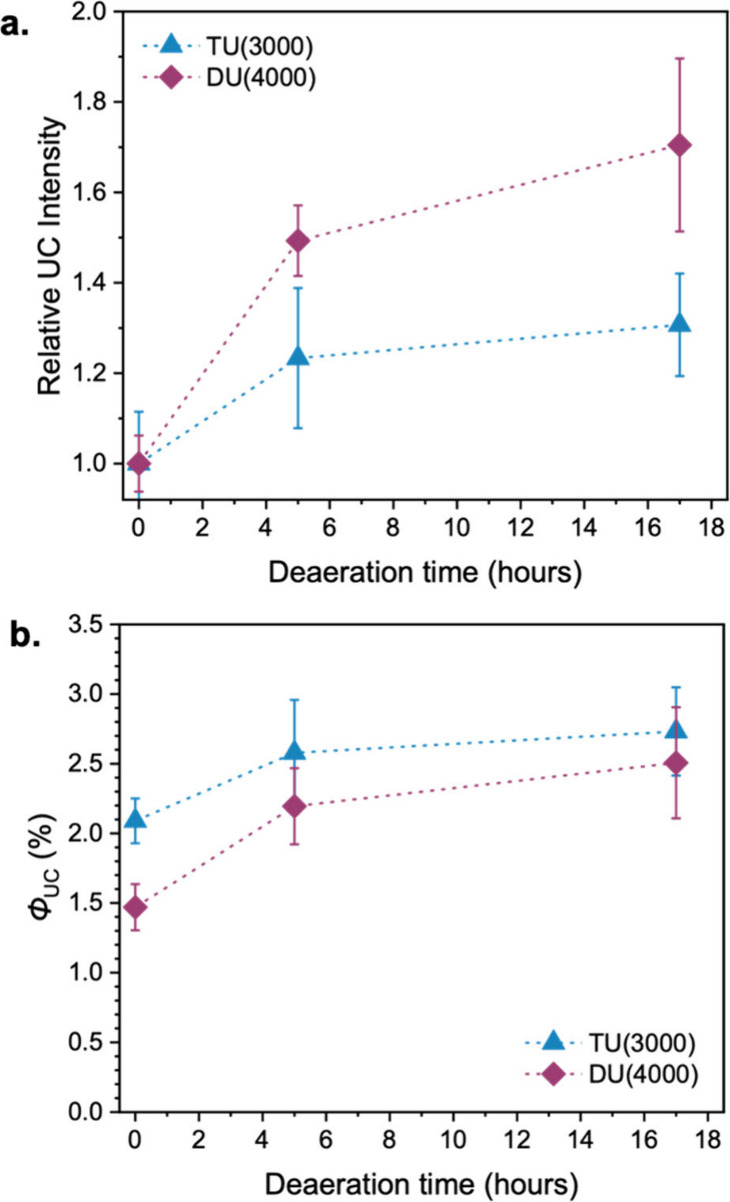
Effect of deaeration on the upconversion efficiency for TU(3000) and DU(4000) samples doped with PdOEP (0.1 mM) and DPA (10 mM). (a) Integrated UC emission intensity (λ_ex_ *=* 532 nm, λ_em_ = 380–500 nm), relative to that without deaeration, as a function of time purged with N_2_. (b) Estimated upconversion quantum yields (Φ_UC_) as a function of the deaeration time. λ_ex_ = 530 nm, 1 W cm^–2^.

## Conclusions

In this study, we have clearly demonstrated a direct relationship between the ureasil host structure and the extent of oxygen quenching of TTA-UC using the PdOEP/DPA chromophore pair. Direct measurement of the oxygen transmission rate through bulk (undoped) ureasils revealed that the oxygen permeability decreased both with increased silica content and with branching of the organic polymer chains. To the best of our knowledge, this is the first in-depth study of the oxygen barrier properties of ureasil materials. This was supported by the local oxygen permeabilities obtained from analysis of the oxygen quenching of the PdOEP phosphorescence in the absence of DPA. For PdOEP/DPA-doped ureasils, kinetic analysis of the phosphorescence decays measured under an N_2_ atmosphere demonstrated faster TTET rates with decreased silica content and branching, but that this was offset by parasitic re-excitation of PdOEP, leading to roughly equal effective TTET efficiencies across all hosts. Analysis of the same system in air suggested that competition between oxygen quenching and TTET exists, with different quenching mechanisms appearing to dominate over irradiation time. Finally, comparison of upconversion emission in the presence and absence of ambient oxygen confirmed that the variation in ambient Φ_UC_ across different ureasil hosts primarily arises from the differing abilities of ureasil hosts to prevent oxygen quenching. This demonstrates that, while long, mobile organic chains facilitate rapid energy transfer steps, tuning of the silica content to minimize oxygen permeation is crucial when optimizing TTA-UC in ureasil and similar hybrid hosts. In future work, we plan to apply this knowledge in the design of new ureasil-based materials with improved oxygen barrier properties and enhanced ambient upconversion performance. More widely, this work demonstrates the importance of evaluating the oxygen barrier properties of potential hosts for solid-state TTA-UC and establishes a precedent for their characterization. Through this, effective host materials can be developed that remove the need for deoxygenation of TTA-UC systems, improving the scalability and practicality of these systems for real-world applications.

## Experimental Methods

### Fabrication of Ureasil Organic–Inorganic Hybrids

Ureasil samples were fabricated via a two-step sol–gel process as described previously.^53^ First, Jeffamine was dissolved in THF (0.35 M for D-2000 or T-3000, 0.175 M for D-4000), and ICPTES added in a molar ratio of 2:1 for bis-functionalized amines and 3:1 for tris-functionalized amines. The reaction mixture was heated under reflux at 70 °C for 24 h, before cooling to room temperature to yield the di- or triureapropyltriethoxysilane (D- and T-UPTES, respectively) precursor in solution. In the second step, the gelling agents, EtOH, HCl (0.5 M) and H_2_O, were sequentially added such that the molar ratio of -Si­(OEt)­3:EtOH:HCl:H_2_O was 176:350:1:265, with thorough mixing after each addition. For D-UPTES based on Jeffamine D-4000, double the quantity of HCl was added to accelerate an otherwise slow gelling process. The resulting mixture was cast into a poly­(propylene) mold covered with an in-built lid or Parafilm M to ensure slow evaporation of excess THF in the samples over 1–2 days, followed by further oven drying at 40 °C for 1–2 days, until all remaining THF had evaporated. The final ureasils were obtained as free-standing monoliths of thickness of ∼1 mm. Where the exact thickness was relevant to the measurement (e.g., for bulk oxygen permeation analysis), this was measured using a micrometer and is quoted alongside the data.

The quantity of D- or T-UPTES used was calculated based on the mold size, and expected final density and shrinkage, such that the final, dry monolith would be 1 mm thick.

For samples doped with chromophores, stock solutions in THF of PdOEP (0.5 mM) and DPA (50 mM) were prepared and added to the D- or T-UPTES solution in the necessary volumes to give the desired concentrations in the final dry monolith prior to the addition of gelling agents. For example, if the expected final volume was 1 cm3, and the desired concentration was 0.1 mM PdOEP, 200 μL of stock solution was added.

### Bulk Oxygen Permeation Measurements

Bulk oxygen permeation measurements were performed using a Model 8101 Oxygen Permeation Analyzer (Systech Illinois). For each host material, two samples, both with a 5 cm^2^ circular mask, were measured in parallel, with three repeat scans. In each scan, the oxygen transmission rate (OTR) at 23 °C from a 100% O_2_ top chamber to a 100% N_2_ bottom chamber was measured at 30 min intervals, alternating between cells, until five consecutive values for each cell fell within 1% of each other. The final value in each case was taken as the OTR.

To normalize for variations in pressure, the angular ratio (OTR) for each scan was converted to a value of permeance:

9 $$\mathrm{permeance}=\frac{\mathrm{OTR}}{{\Delta p}_{O_{2}}}$$

where the partial pressure change between chambers, Δ*p*_O_2__, was equal to the recorded atmospheric pressure. From the average permeance over three scans, the oxygen permeability coefficient *P*_O_2__ could be calculated using thickness *t* of the sample (averaged over five random points):

10 $$P_{O_{2}}=\mathrm{permeance}\times t$$

The values of *P*_O_2__ quoted are the averages over the two samples. The errors quoted are either the error in the mean oxygen permeability coefficient, or the compound error arising from the respective errors in the mean permeance and mean thickness for each sample, whichever is greater.

### Time-Resolved Photoluminescence Measurements

Photoluminescence decay measurements were performed using the multichannel scaling (MCS) method on a FLS 1000 TCSPC spectrometer (Edinburgh Instruments Ltd.). The emission decay was recorded by using a high-speed photomultiplier tube (PMT-980) equipped with TCC2 counting electronics. The phosphorescence decay profiles were measured at an emission wavelength of 665 nm using 532 nm laser excitation (MGL-III-532 laser) and a visible PMT-980 detector, with long-pass filter (cutoff 550 nm) applied in front of the detector. For upconversion decays, the emission wavelength was changed to 440 nm, and the filter in front of the detector changed to a short-pass filter (cutoff 500 nm). The pulse repetition rate, collection range, and pulse width were adjusted to best capture the decay profile but kept consistent for equivalent measurements on different samples. The detector bandwidth was adjusted between 1 and 5 nm to give a count rate around 10000 counts per second. The instrument response function (IRF) was measured using with a SiO2 particle suspension solution (Ludox colloidal silica) using a neutral density filter (OD = 3) in front of the excitation source and with no emission filter. IRF scans were measured immediately after the corresponding decays using the same instrument parameters, except with adjustment of the detector to 532 nm and a bandwidth of 3 nm.

### Upconversion Intensities and Quantum Yields

The UC emission intensities and quantum yields of all samples were measured with an FLS 1000 TCSPC spectrometer (Edinburgh Instruments Ltd.). Samples were excited with a 532 nm laser (MGL-III-532, 200 mW). The laser power was adjusted to 1 W cm^–2^ using a Thorlabs PM100A Power Meter Console combined with a S120VC Si photodiode power sensor (range: 200–1100 nm). All spectra were measured with an integrating sphere (SNS125 5 in. sphere, three windows, International Light Technologies).

For all UC emission measurements, including those used to calculate the quantum yield, the detector bandwidth was 0.5 nm, measuring from 380 to 500 nm with a step size of 1 nm and a dwell time of 0.1 s, and a short-pass filter (cutoff 500 nm, Thorlabs) placed in front of the detector. For all measurements of scattered laser intensity, the detector bandwidth was 0.5 nm, measuring from 530 to 534 nm with a step size of 0.1 nm and a dwell time of 0.1 s, and a neutral density filter (OD = 3) was placed in front of the excitation source. All spectra were taken with three averaged scans and corrected for the transmission of the relevant filters in the relevant wavelength range.

For emission measurements as a function of deaeration, samples were cut to a size of 1.2 × 2 cm and placed inside a sealed cuvette, before flowing with N_2_. While the N_2_ flow rate is not known, both samples were flowed in parallel and so should have experienced the same flow rate for the same length of time. The same samples of each host were used for all measurements, measured twice at each deaeration time, rotating 180° between measurements to allow irradiation of a different spot.

For upconversion quantum yield measurements, the samples were cut into a 1 × 1 cm square and loaded at the center of the sphere by a sample holder. A baffle is placed in front of the observation window, which blocks any scattering and reflection of the laser from the sample surface. The angle of the sample holder is adjustable. The normal direction of the sample holder is 45° to the excitation beamline.

## Supplementary Material



## Notes

Materials and methods: materials, fitting of photoluminescence decays, calculation of upconversion quantum yields; derivation of Stern–Volmer analysis for calculating oxygen permeability; supporting data from bulk oxygen permeation measurements, fitting of time-resolved phosphorescence and upconversion emission, calculation of TTET efficiency (PDF)
