# Supplementary material for: Interplay between Host Structure, Oxygen Quenching, and Triplet–Triplet Annihilation Upconversion in Hybrid Polymer Hosts
Source: ACS Appl Opt Mater. 2026 Apr 8;4(4):1169–79. doi: 10.1021/acsaom.6c00025 (PMC13123409; doi:10.1021/acsaom.6c00025)
Supplement: Supplementary file 1 [file ot6c00025_si_001.pdf]

Supporting Information

Interplay between host structure, oxygen quenching and triplet-triplet annihilation upconversion in hybrid polymer hosts

*Georgina H. Burgoyne Morris[a], Larissa Gomes Franca[a], Abigail R. Collins[a], and Rachel C. Evans[a]**

[a] Department of Materials Science & Metallurgy, University of Cambridge, 27 Charles Babbage Road, Cambridge, CB3 0FS, UK

* Corresponding Author: rce26@cam.ac.uk

TABLE OF CONTENTS

1. Materials and methods 2

1.1. Materials 2

1.2. Fitting of photoluminescence decays 2

1.3. Calculation of upconversion quantum yields 3

2. Derivation for calculation of oxygen permeability coefficient from Stern-Volmer analysis 5

3. Supporting data 6

3.1. Bulk oxygen permeation measurements 6

3.2. Phosphorescence lifetimes: PdOEP-doped ureasils 7

3.3. Phosphorescence lifetimes: TTA-UC pair-doped ureasils 13

3.4. Upconversion lifetimes: TTA-UC pair-doped ureasils 20

4. TTET Efficiencies: TTA-UC pair-doped samples 21

5. References 22

1. Materials and methods

1.1. Materials

Tetrahydrofuran (THF, ≥99.9%), ethanol (95.0%), hydrochloric acid (37%) and 3-(triethoxysilyl)propylisocyanate (ICPTES, 95.0%) were purchased from Fisher Scientific. 9,10-Diphenylanthracene (DPA, 99%) was purchased from Alfa Aesar. 2,3,7,8,12,13,17,18-Octaethyl-21H,23H-porphine palladium (II) (PdOEP, 85%) was purchased from Sigma-Aldrich. Water was obtained from a Millipore Simpak 2 water purification system. All chemicals were used as received.

JEFFAMINE® D-X and T-X (D denotes bis-end functionality, t denotes tris-end functionality, and X is representative of the molecular weight, MW): poly(propylene glycol) bis(2-aminopropyl ether) (JEFFAMINE® D-2000, MW = 2000 g mol^{-1}) was purchased from Sigma-Aldrich; poly(propylene glycol) bis(2-aminopropyl ether) (JEFFAMINE® D-4000, MW = 4000 g mol^{-1}) and glyceryl poly(oxypropylene) triamine (JEFFAMINE® T-3000, MW = 3000 g mol^{-1}) were provided as a kind gift from Huntsman.

1.2. Fitting of photoluminescence decays

Individual tail-fits were applied to each emission decay curve using the FAST software package (Edinburgh Instruments) using a multiexponential decay function:

$$I(t) = \sum_{i}^{n} \alpha_i e^{-t/\tau_\mathrm{i}} \quad \text{(Eq.S1)}$$

where α_i and τ_i are the amplitude and lifetime of the *i*th component, respectively, and α_i is normalised to unity for *n* components. The goodness of fit was assessed using the reduced chi-square statistics, χ^2, and the randomness of the residuals.[1] The fractional contributions (f_i) are also reported, where

$$f_i = \frac{\alpha_i \tau_i}{\sum_j^n \alpha_j \tau_j} \quad \text{(Eq.S2)}$$

From this fit, the integrated decay intensity (*I*) was calculated:

$$I = \sum_{i}^{n} \alpha_i \tau_i \quad \text{(Eq.S3)}$$

For upconversion decays, the average lifetime (<τ>) was calculated:

$$\langle \tau \rangle = \sum_{i}^{n} f_i \tau_i \quad \text{(Eq.S4)}$$

Lifetime fits for each sample have been reported, along with the errors in fitted quantities where physically meaningful. For measurements used in quantitative analysis, repeats were taken over 3 samples. Overall values and errors reported for fitted quantities are from the mean across the repeats and are propagated for subsequent calculated values.

1.3. Calculation of upconversion quantum yields

The upconversion quantum yield (Φ_{UC}) was calculated for each sample using the experimental approach described by Porrès et al.[2] and the following formulae:[3,4]

$$\Phi_{\text{UC}} = \frac{E_{x,\,\text{in}} - (1 - A)E_{x,\,\text{out}}}{AL_{b,\,\text{in}}} \quad \text{(Eq.S5)}$$

where A is the fraction of the photons absorbed directly by the sample, which is corrected by removing the secondary absorption from the sphere-reflected photons:

$$A = \frac{L_{x,\,\text{out}} - L_{x,\,\text{in}}}{L_{x,\,\text{out}}} \quad \text{(Eq.S6)}$$

where E is the integrated photon counts from emission spectra, and L is the integrated photon counts from the scattering spectra. x delineates measurements with the sample present, while b indicates measurements with an empty sample holder. "in" means the sample was in the path of the excitation beam, facing towards both the excitation window and the observation window, and "out" indicates the sample is moved out of the beam line by rotating the sample holder 180° such that its back faces the windows. The empty-sphere data sets were collected at the beginning of the measurement, under the same conditions of the sample-in measurement and were shared in all calculations of samples measured in the same hour-long period. During the calculation, all data were corrected by the transmittance of each filter used, and normalised based on the slit-width, scan step and the scan duration used. A quantum yield is defined as the

ratio of absorbed to emitted photons, meaning the upconversion quantum yield is limited to 50% since this is a bimolecular process. While some papers report this as a normalised value, Φ_{UC} is reported to its un-normalised value here, to a maximum of 50%.[5]

All values of Φ_{UC} in air are quoted as an average over multiple samples, with the standard error in the mean. Values of Φ_{UC} after deaeration are predicted by multiplying the Φ_{UC} in air by the ratio of UC intensity after deaeration to that before, and the errors quoted are the compound errors of those for each integrated intensity measurement and the quantum yield in air.

2. Derivation for calculation of oxygen permeability coefficient from Stern-Volmer analysis

The use of Stern-Volmer and Freundlich analyses of phosphorescence kinetics data is based on those used by Douglas & Eaton.[6] For diffusion-controlled quenching by a constant concentration of oxygen, the Stern-Volmer equation states that:

$$\frac{I_0}{I} = \frac{\tau_0}{\tau} = 1 + \tau_0 k_Q [O_2] \qquad \text{(Eq.S7)}$$

where I and I_0 are the emission intensities in the presence and absence, respectively, of oxygen at concentration $[O_2]$, and τ and τ_0 are the equivalent lifetimes. The quenching rate constant, k_Q, can be assumed to be equal to the product of the quenching efficiency factor, f_Q, and the diffusion-controlled bimolecular, k_{diff}, which in turn is given by:

$$k_{\mathrm{diff}} = N_A 4\pi (r_L + r_{\mathrm{O_2}}) D_{\mathrm{O_2}} \qquad \text{(Eq.S8)}$$

where N_A is Avogadro's number, r_L and $r_{\mathrm{O_2}}$ are the molecular radii of the luminophore and oxygen, respectively, and $D_{\mathrm{O_2}}$ is the diffusivity of oxygen in the matrix.

Meanwhile, for a material in equilibrium with oxygen at partial pressure $p_{\mathrm{O_2}}$, the internal concentration of dissolved oxygen is given by:

$$[O_2] = S_{\mathrm{O_2}} p_{\mathrm{O_2}} \qquad \text{(Eq.S9)}$$

where $S_{\mathrm{O_2}}$ is the solubility of oxygen in the material. Combining equations S7-S9 gives:

$$\frac{I_0}{I} = \frac{\tau_0}{\tau} = 1 + \tau_0 f_Q N_A 4\pi (r_L + r_{\mathrm{O_2}}) D_{\mathrm{O_2}} S_{\mathrm{O_2}} p_{\mathrm{O_2}} \qquad \text{(Eq.S10)}$$

Since, by definition, the oxygen permeability coefficient, $P_{\mathrm{O_2}}$, is equal to the product of solubility and diffusivity (*i.e.* $P_{\mathrm{O_2}} = S_{\mathrm{O_2}} D_{\mathrm{O_2}}$), equation S10 can be rewritten as in the main text:

$$\frac{I_0}{I} = \frac{\tau_0}{\tau} = 1 + K_{\mathrm{SV}}^{\mathrm{gas}} p_{\mathrm{O_2}} \qquad \text{(Eq.S11)}$$

$$K_{\mathrm{SV}}^{\mathrm{gas}} = \tau_0 f_Q N_A 4\pi (r_L + r_{\mathrm{O_2}}) P_{\mathrm{O_2}} \qquad \text{(Eq.S12)}$$

3. Supporting data

3.1. Bulk oxygen permeation measurements

Table S1. Results of bulk oxygen permeation measurements for undoped ureasil samples. Parameters are as defined in the main manuscript.

Ureasil	Sample	Permeance (gpu[a])	t (mm)	P_{O_2} (barrer[b])	Average P_{O_2} (barrer[b])
DU(4000)	1	0.0273 ± 0.0002	1.00 ± 0.01	27.3 ± 0.5	26.6 ± 0.7
	2	0.0269 ± 0.0002	0.97 ± 0.02	25.9 ± 0.6	
DU(2000)	1	0.01875 ± 0.00007	0.71 ± 0.03	13.4 ± 0.5	13.4 ± 0.5
	2	Not measured[c]	-	-	
TU(3000)	1	0.01411 ± 0.00005	0.83 ± 0.03	11.7 ± 0.4	11.6 ± 0.3
	2	0.01285 ± 0.00005	0.89 ± 0.03	11.5 ± 0.4	

[a]Gas permeation units. 1 gpu =10^{-6} $cm^3_{STP}/cm^2 \cdot s \cdot cmHg$
[b]1barrer= 10^{-12} $cm^3_{STP} \cdot cm/cm^2 \cdot s \cdot cmHg$
[c]Measurement of the oxygen permeation for the second DU(2000) sample failed due to damage to the sample, and could not be repeated due to issues with the instrument

3.2. Phosphorescence lifetimes: PdOEP-doped ureasils

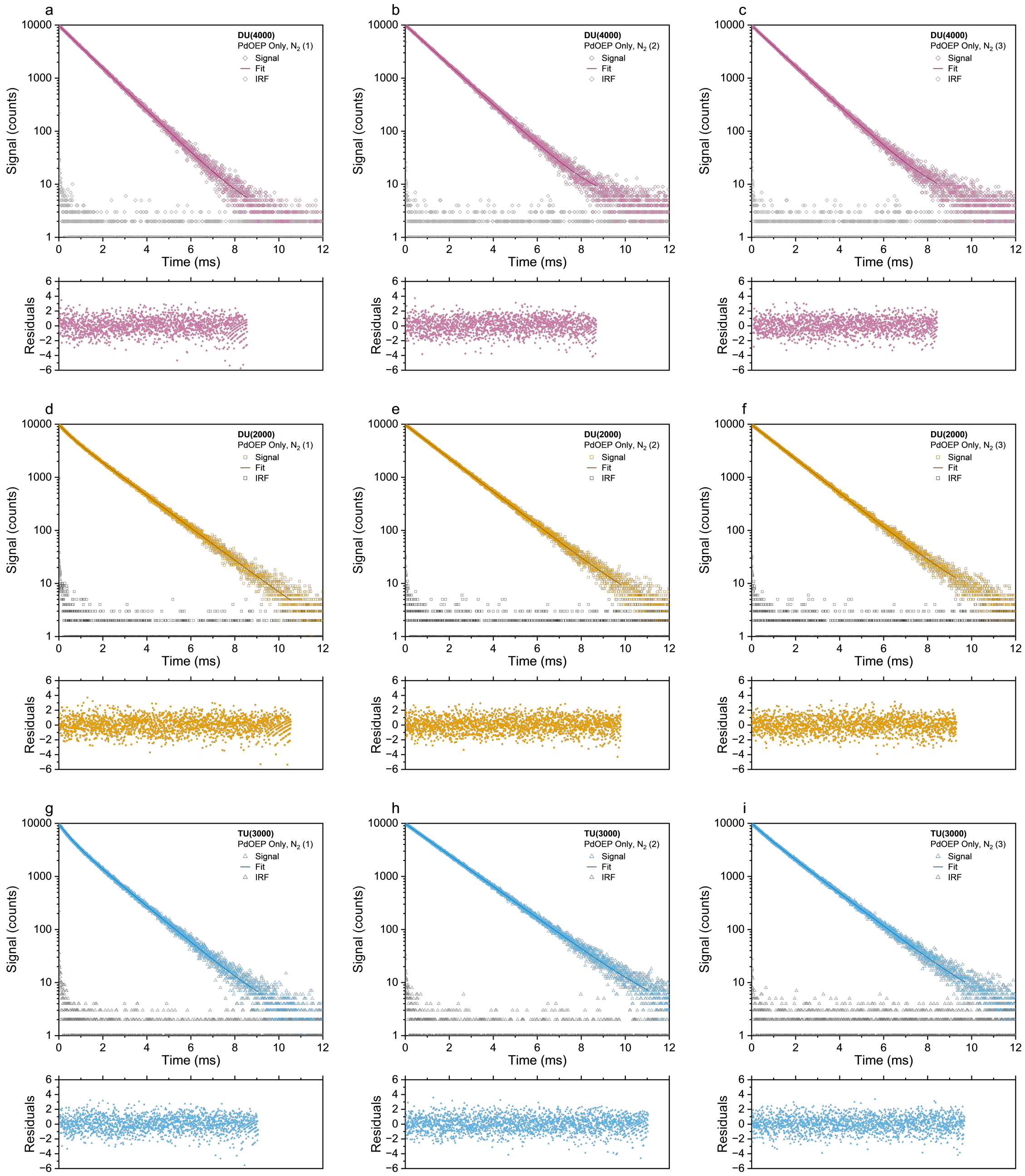

Figure S1. Phosphorescence decays and IRF (λ_{ex} = 532 nm, λ_{em} = 665 nm), and corresponding fits and residuals, for PdOEP (0.1 mM) in three samples of DU(4000) (a-c), DU(2000) (d-f) and TU(3000) (g-i), after purging in a sealed cuvette with N_2 for 4 hours.

Table S2. Multiexponential fitting parameters for phosphorescence decays (λ_{ex} = 532 nm, λ_{em} = 665 nm) of PdOEP (0.1 mM) in ureasil hosts, measured in a sealed cuvette after purging with N_2 for 4 hours.

Ureasil	Sample	χ^2 [a]	i	α_i [b]	f_i (%) [c]	τ_i (μs) [d]	I_0 [e]
DU(4000)	1	1.291	0[f]	1[h]	100[h]	1100[h]	1100[h]
	2	1.149	0	1[h]	100[h]	1200[h]	1200[h]
	3	1.086	0	0.90[h]	93 ± 13	1200[h]	1160 ± 180
			res[g]	0.10[h]	7 ± 9	810[h]	
DU(2000)	1	1.189	0	0.79[h]	91.9 ± 0.5	1400[h]	1200 ± 7
			res	0.21[h]	8.1 ± 0.2	460[h]	
	2	1.087	0	1[h]	100[h]	1400[h]	1400[h]
	3	1.140	0	1[h]	100[h]	1300[h]	1300[h]
TU(3000)	1	1.198	0	0.67[h]	85.3 ± 0.7	1300[h]	1016 ± 7
			res	0.33[h]	14.7 ± 0.2	440[h]	
	2	1.143	0	1[h]	100[h]	1500[h]	1500[h]
	3	1.160	0	0.85[h]	93.5 ± 0.9	1400[h]	1016 ± 7
			res	0.15[h]	6.5 ± 0.4	560[h]	1276 ± 13

[a] Chi-squared, a measure of goodness of fit. [b] Normalized pre-exponential factor for *i*th component. [c] Fractional contribution of the ith component. [d] Lifetime of the *i*th component. [e] Integrated intensity. [f] Indicative of unquenched lifetime. [g] Indicative of lifetime arising from quenching by residual oxygen quenching. [h] The errors generated were not physically meaningful, so have not been quoted

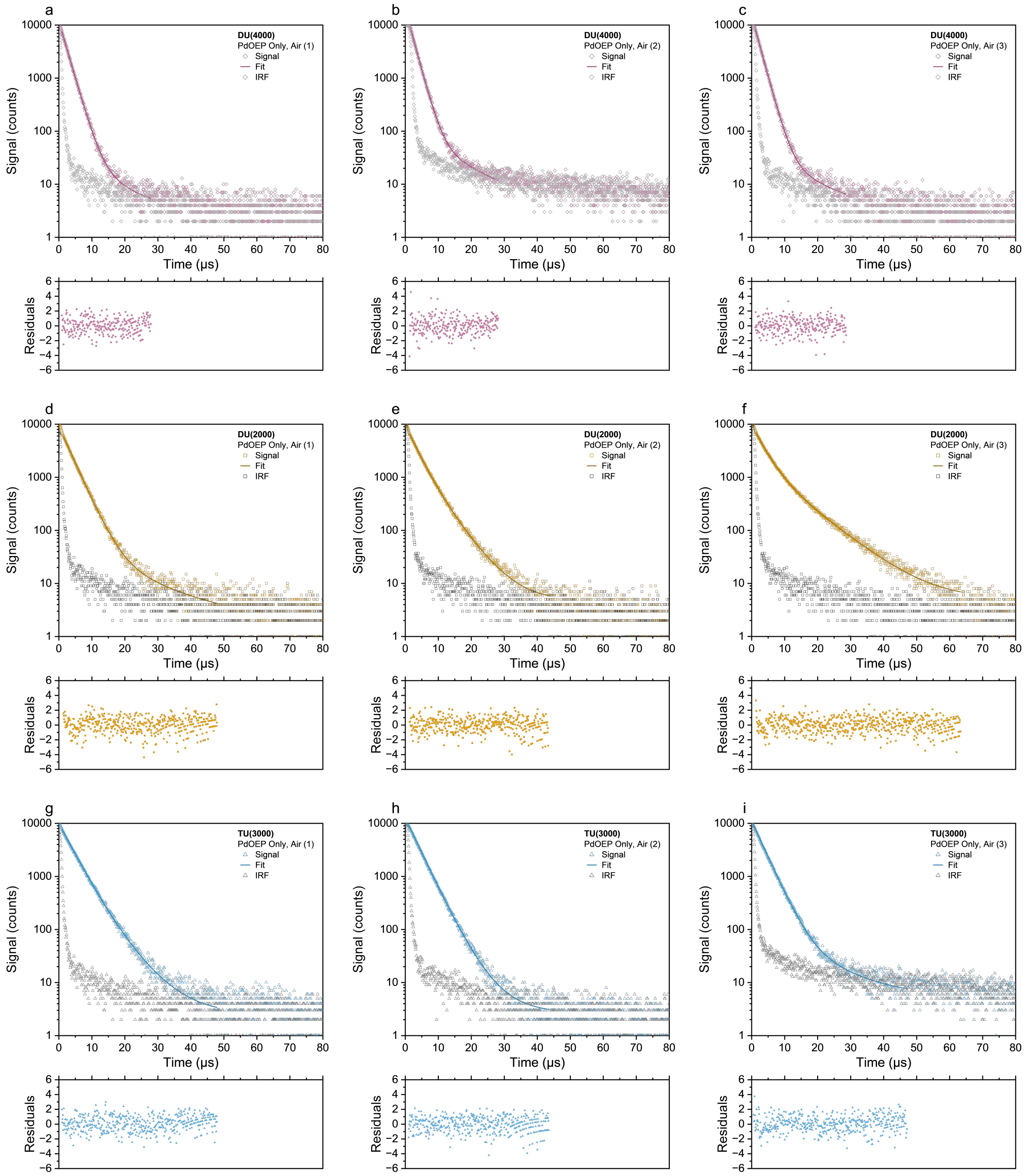

Figure S2. Phosphorescence decays and IRF (λ_{ex} = 532 nm, λ_{em} = 665 nm), and corresponding fits and residuals, for PdOEP (0.1 mM) in three samples of DU(4000) (a-c), DU(2000) (d-f) and TU(3000) (g-i), measured in air.

Table S3. Multiexponential fitting parameters for phosphorescence decay curves (λ_{ex} = 532 nm, λ_{em} = 665 nm) of PdOEP (0.1 mM) in ureasil hosts, measured in air.

Ureasil	Sample	χ^2 [a]	i	α_i [b]	f_i (%)[c]	$\tau_{air,i}$ (μs)[d]	I_{air}[e]
DU(4000)	1	1.051	1	0.994[f]	97.3 ± 0.3	2.02[f]	2.065 ± 0.014
			2	0.006[f]	2.7 ± 0.6	9.18[f]	
	2	1.266	1	0.99[f]	94.9 ± 0.3	187[f]	1.945 ± 0.013
			2	0.01[f]	5.1 ± 0.6	9.73[f]	
	3	1.110	1	0.994[f]	97.0 ± 0.3	2.02[f]	1.934 ± 0.012
			2	0.006[f]	3.0 ± 0.5	9.54[f]	
DU(2000)	1	1.187	1	0.99[f]	95.6 ± 0.3	3.11[f]	3.22 ± 0.02
			2	0.01[f]	4.4 ± 0.6	13.7[f]	
	2	1.110	1	0.76[f]	62 ± 3	2.95[f]	3.6 ± 0.2
			2	0.24[f]	38 ± 5	5.69[f]	
	3	1.041	1	0.76[f]	52.4 ± 0.4	3.14[f]	4.56 ± 0.07
			2	0.24[f]	47.6 ± 1.4	9.05[f]	
TU(3000)	1	1.114	1	0.92[f]	85.8 ± 1.4	3.58[f]	3.85 ± 0.13
			2	0.08[f]	14 ± 3	7.00[f]	
	2	1.118	1	0.97[f]	94 ± 2	3.24[f]	3.34 ± 0.13
			2	0.03[f]	6 ± 3	6.43[f]	
	3	1.176	1	0.99[f]	95.4 ± 0.2	3.14[f]	3.25 ± 0.02
			2	0.01[f]	4.6 ± 0.6	11.8[f]	

[a] Chi-squared, a measure of goodness of fit. [b] Normalized pre-exponential factor for *i*th component. [c] Fractional contribution of the ith component. [d] Lifetime of the *i*th component. [e] Integrated intensity. [f] The errors generated were not physically meaningful, so have not been quoted

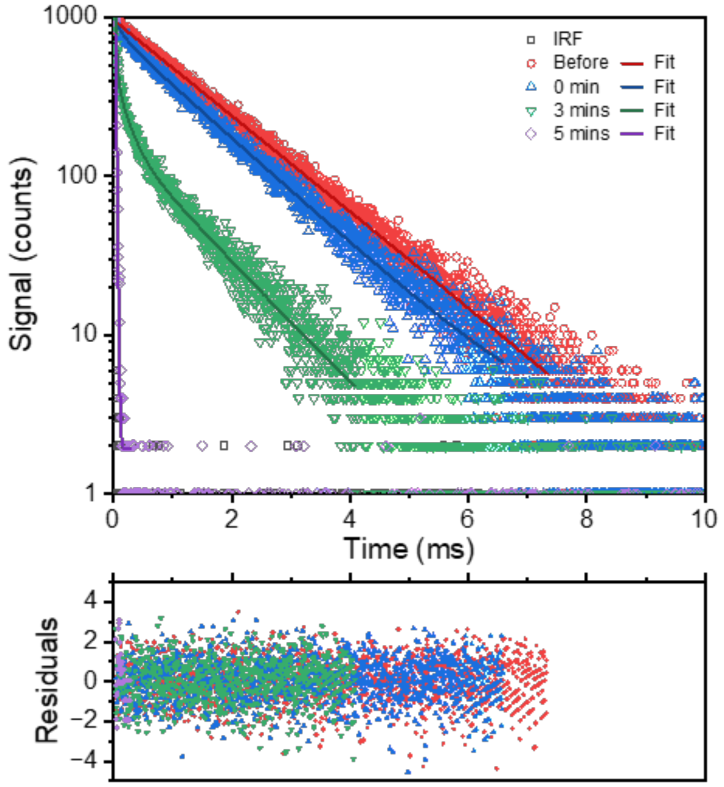

Figure S3. Phosphorescence decays and IRF (λ_{ex} = 532 nm, λ_{em} = 665 nm) of PdOEP (0.1 mM) in TU(3000). The sample was first deaerated by purging a sealed cuvette with N_2 for 4 hours (before, red circles) before gradually being exposed to air for different times.

Table S4. Multiexponential fitting parameters for phosphorescence decay curves (λ_{ex} = 532 nm, λ_{em} = 665 nm) of PdOEP (0.1 mM) in TU(3000). The sample was first deaerated by purging a sealed cuvette with N_2 for 4 hours before gradually being exposed to air for different times.

Exposure Time (min)	$\chi^{2\,a}$	*i*	a_i [b]	f_i (%)[c]	τ_i (μs)[d]
Before	1.151	1	1[f]	100[f]	1400[f]
0	1.117	1	0.14[f]	2.2 ± 0.2	180[f]
		2	0.86[f]	97.8 ± 0.8	1300[f]
3	1.209	1	0.62[f]	6.7 ± 0.3	24.2[f]
		2	0.23[f]	20.2 ± 1.0	200[f]
		3	0.15[f]	73.1 ± 1.9	1100[f]
5	2.106[e]	1	1[f]	100[f]	12.7[f]

[a] Chi-squared, a measure of goodness of fit. [b] Normalized pre-exponential factor for *i*th component. [c] Fractional contribution of the ith component. [d] Lifetime of the *i*th component. [e] Achieving a good fit was not possible due to the small number of data points available. [f] The errors generated were not physically meaningful, so have not been quoted

Table S5. Calculation of oxygen permeability coefficients through three different photophysical analytical methods

Ureasil	Stern-Volmer Intensity Analysis[a]		Freundlich Intensity Analysis[a]		Stern-Volmer Lifetime Analysis[a]	
	K_{SV}^{gas} (10^{-3} Pa^{-1})[a]	P_{O_2} (barrer)[a]	K_{F}^{gas} (10^{-3} Pa^{-1})[a]	P_{O_2} (barrer)	K_{SV}^{gas} (10^{-3} Pa^{-1})	P_{O_2} (barrer)
DU(4000)	27.3 ± 0.9	16.8 ± 0.7	38.2 ± 0.6	23.5 ± 0.8	28.4 ± 1.1	17.5 ± 0.4
DU(2000)	16.1 ± 1.8	8.4 ± 1.0	21.8 ± 1.3	11.5 ± 0.7	20.9 ± 0.7	11.0 ± 0.2
TU(3000)	17.0 ± 2.0	8.7 ± 1.1	23.2 ± 1.5	11.9 ± 0.9	19.8 ± 1.1	10.1 ± 0.4

[a] Defined in main manuscript.

3.3. Phosphorescence lifetimes: TTA-UC pair-doped ureasils

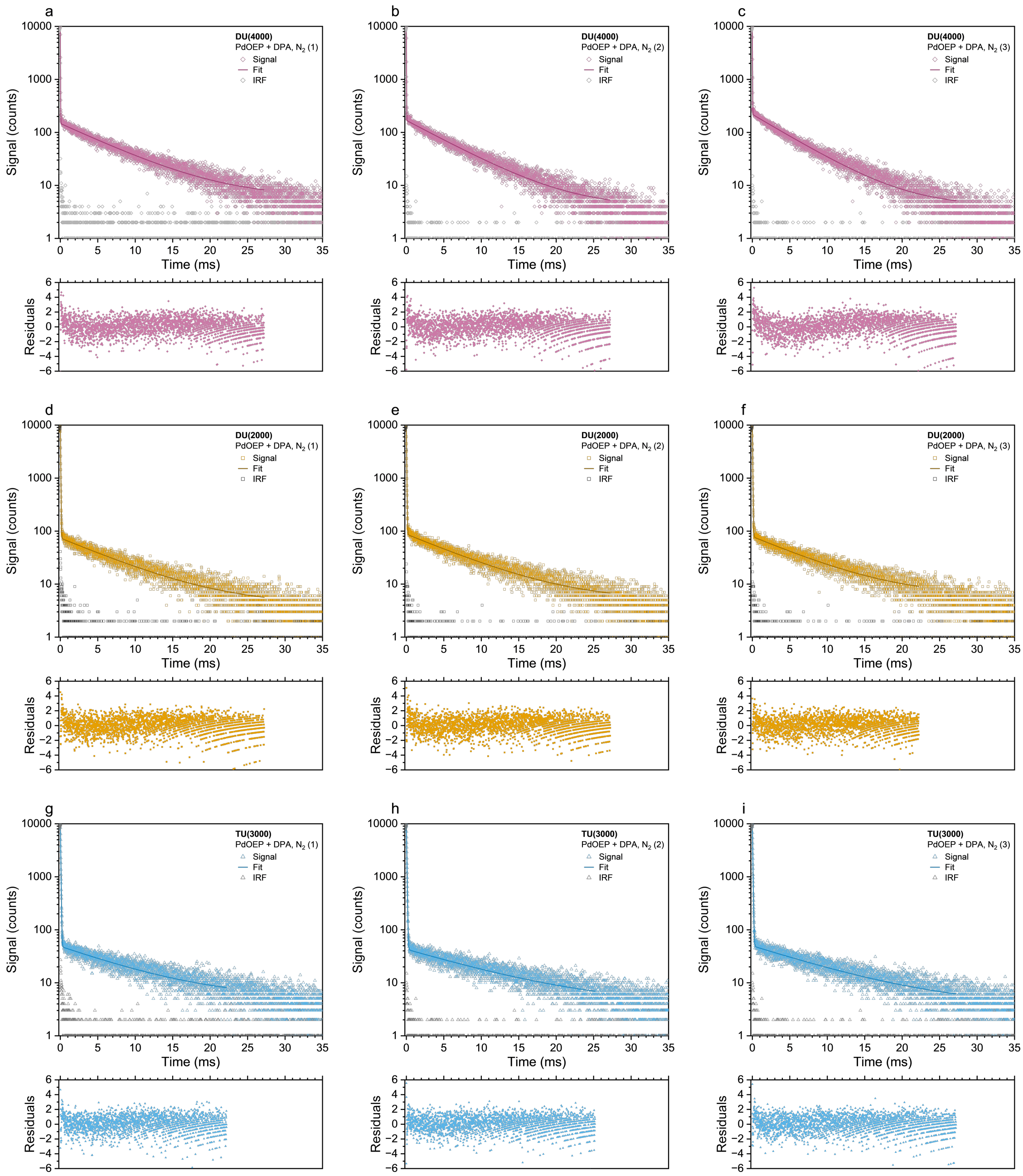

Figure S4. Phosphorescence decays and IRF (λ_{ex} = 532 nm, λ_{em} = 665 nm), and corresponding fits and residuals, for PdOEP (0.1 mM), in the presence of DPA (10 mM), in three samples of DU(4000) (a-c), DU(2000) (d-f) and TU(3000) (g-i), measured in a sealed cuvette after purging with N_2 for 4 hours.

Table S6. Multiexponential fitting parameters for phosphorescence decays (λ_{ex} = 532 nm, λ_{em} = 665 nm) of PdOEP (0.1 mM), in the presence of DPA (10 mM), in ureasil hosts, measured in a sealed cuvette after purging with N_2 for 4 hours.

Ureasil	Sample	χ^{2} [a]	i	α_i [b]	f_i (%)[c]	$\tau_{S+E,i}$ (μs)[d]	I_{S+E}[e]
DU(4000)	1	1.538	1	0.994[f]	24.5 ± 0.4	14[f]	55.1 ± 0.3
			2	0.006[f]	75.5 ± 0.5	6600[f]	
	2	1.588	1	0.993[f]	23.5 ± 0.3	12[f]	52.1 ± 0.3
			2	0.007[f]	76.5 ± 0.4	5700[f]	
	3	1.726	1	0.991[f]	20.9 ± 0.3	13[f]	60.6 ± 0.3
			2	0.009[f]	79.1 ± 0.4	5100[f]	
DU(2000)	1	1.390	1	0.995[f]	45.4 ± 0.3	30[f]	66.3 ± 0.3
			2	0.005[f]	54.6 ± 0.4	7200[f]	
	2	1.346	1	0.993[f]	37.7 ± 0.3	32[f]	82.8 ± 0.4
			2	0.007[f]	62.3 ± 0.4	7200[f]	
	3	1.341	1	0.994[f]	41.9 ± 0.3	33[f]	77.3 ± 0.4
			2	0.006[f]	58.1 ± 0.4	7000[f]	
TU(3000)	1	1.400	1	0.997[f]	59.5 ± 0.4	41[f]	67.9 ± 0.4
			2	0.003[f]	40.5 ± 0.4	8200[f]	
	2	1.303	1	0.997[f]	60.8 ± 0.3	46[f]	75.4 ± 0.4
			2	0.003[f]	39.2 ± 0.4	9900[f]	
	3	1.341	1	0.996[f]	57.5 ± 0.3	45[f]	78.7 ± 0.4
			2	0.004[f]	42.5 ± 0.4	9300[f]	

[a] Chi-squared, a measure of goodness of fit. [b] Normalized pre-exponential factor for *i*th component. [c] Fractional contribution of the ith component. [d] Lifetime of the *i*th component. [e] Integrated intensity. [f] The errors generated were not physically meaningful, so have not been quoted.

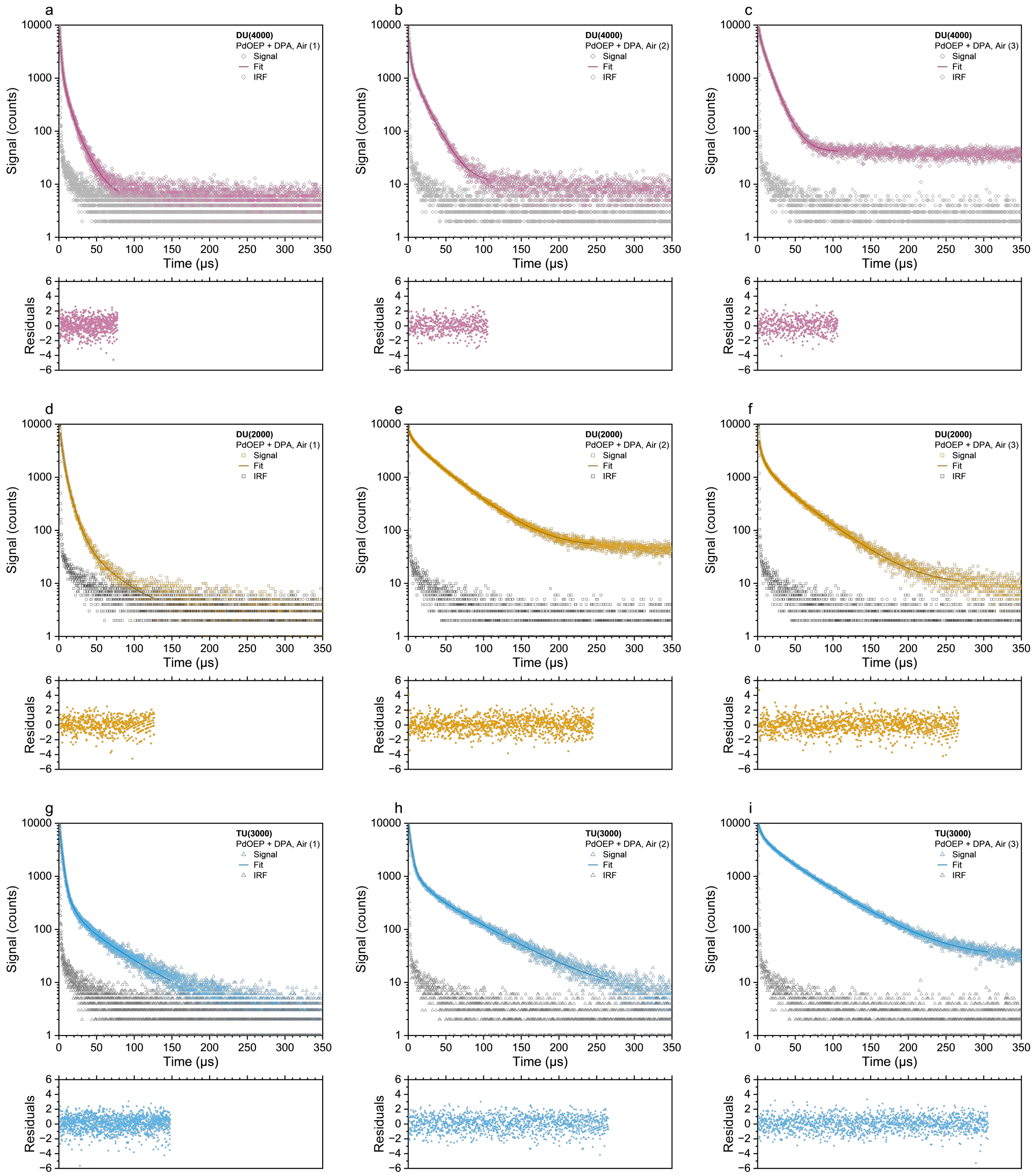

Figure S5. Phosphorescence decays and IRF (λ_{ex} = 532 nm, λ_{em} = 665 nm), and corresponding fits and residuals, for PdOEP (0.1 mM), in the presence of DPA (10 mM), in three samples of DU(4000) (a-c), DU(2000) (d-f) and TU(3000) (g-i), measured in air

Table S7. Multiexponential fitting parameters for phosphorescence decays (λ_{ex} = 532 nm, λ_{em} = 665 nm) of PdOEP (0.1 mM), in the presence of DPA (10 mM), in ureasil hosts, measured in air.

Ureasil	Sample	χ^2 [a]	i	α_i [b]	f_i (%)[c]	$\tau_{S+E,air,i}$ (μs)[d]
DU(4000)	1	1.090	1	0.81[e]	44.4 ± 0.4	1.55[e]
			2	0.16[e]	34.6 ± 1.0	6.24[e]
			3	0.04[e]	21 ± 3	15.3[e]
	2	0.963	1	0.62[e]	17.8 ± 0.6	1.59[e]
			2	0.15[e]	16.5 ± 1.6	5.98[e]
			3	0.23[e]	66 ± 5	15.6[e]
	3	1.02	1	0.29[e]	6.4 ± 0.4	1.74[e]
			2	0.39[e]	38 ± 7	7.56[e]
			3	0.32[e]	55 ± 14	13.3[e]
DU(2000)	1	1.107	1	0.68[e]	40.8 ± 1.0	2.92[e]
			2	0.30[e]	49 ± 3	7.98[e]
			3	0.02[e]	10.2 ± 1.1	31.4[e]
	2	1.050	1	0.20[e]	1.6 ± 0.1	1.94[e]
			2	0.21[e]	8.9 ± 0.4	10.8[e]
			3	0.59[e]	89.5 ± 1.0	37.9[e]
	3	1.142	1	0.47[e]	8.1 ± 0.2	2.52[e]
			2	0.27[e]	21.3 ± 0.8	11.4[e]
			3	0.26[e]	70.6 ± 1.5	40.6[e]
TU(3000)	1	1.113	1	0.88[e]	53.3 ± 0.4	2.88[e]
			2	0.09[e]	18.5 ± 1.4	9.53[e]
			3	0.03[e]	28.2 ± 0.8	44.0[e]
	2	1.111	1	0.83[e]	32.6 ± 0.2	3.12[e]
			2	0.10[e]	16.9 ± 0.8	13.8[e]
			3	0.07[e]	50.5 ± 1.4	54.9[e]

	3	1.018	1	0.36[e]	5.6 ± 0.2	4.05[e]
			2	0.23[e]	14.9 ± 0.5	16.8[e]
			3	0.42[e]	79.5 ± 1.4	48.8[e]

[a] Chi-squared, a measure of goodness of fit. [b] Normalized pre-exponential factor for *i*th component. [c] Fractional contribution of the ith component. [d] Lifetime of the *i*th component. [e] The errors generated were not physically meaningful, so have not been quoted

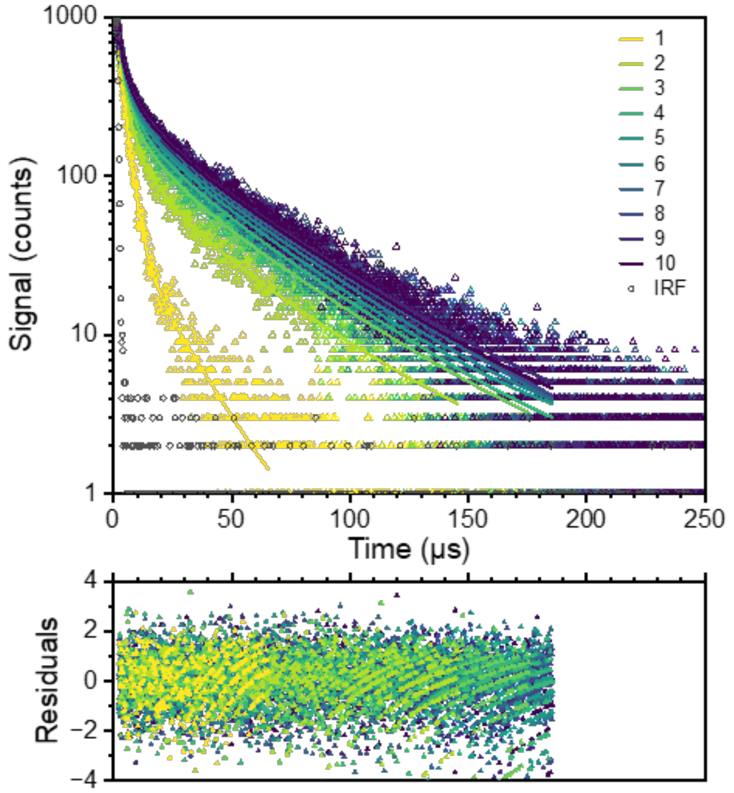

Figure S6. Phosphorescence decays and IRF (λ_{ex} = 532 nm, λ_{em} = 665 nm) of PdOEP (0.1 mM) in the presence of DPA (10 mM) in TU(3000), measured over 10 successive scans, showing the change in lifetime with acquisition time.

Table S8. Multiexponential fitting parameters for phosphorescence decay curves (λ_{ex} = 532 nm, λ_{em} = 665 nm) of PdOEP (0.1 mM) in the presence of DPA (10 mM) in TU(3000), for 10 successive scans.

Scan	χ^2 [a]	i	a_i [b]	f_i (%)[c]	τ_i (µs)[d]
1	1.215	1	0.93 ± 0.01	69 ± 1	2.95[e]
		2	0.07 ± 0.01	31 ± 3	17.1[e]
2	1.060	1	0.71 ± 0.04	23 ± 1	2.92[e]
		2	0.16 ± 0.03	20 ± 3	11.6[e]
		3	0.13 ± 0.03	57 ± 14	40.2[e]
3	1.273	1	0.63 ± 0.04	15.3 ± 0.9	2.94[e]
		2	0.19 ± 0.03	20 ± 3	12.4[e]
		3	0.18 ± 0.03	65 ± 9	42.9[e]
4	1.228	1	0.62 ± 0.04	16 ± 1	3.49[e]
		2	0.16 ± 0.03	17 ± 3	14.7[e]
		3	0.22 ± 0.05	67 ± 14	42.3[e]
5	1.156	1	0.50 ± 0.04	8.6 ± 1	2.44[e]
		2	0.23 ± 0.04	16 ± 3	9.76[e]
		3	0.27 ± 0.02	75 ± 5	39.3[e]
6	1.164	1	0.50 ± 0.07	10 ± 1	3.09[e]
		2	0.21 ± 0.06	14 ± 4	10.4[e]
		3	0.29 ± 0.02	76 ± 6	40.9[e]
7	1.232	1	0.53 ± 0.03	10.5 ± 0.7	3.21[e]
		2	0.22 ± 0.03	20 ± 2	14.9[e]
		3	0.24 ± 0.04	69 ± 11	46.3[e]
8	1.060	1	0.52 ± 0.03	9.3 ± 0.6	3.01[e]
		2	0.21 ± 0.02	17 ± 2	13.4[e]
		3	0.27 ± 0.03	73 ± 8	45.3[e]
9	1.001	1	0.47 ± 0.03	7.1 ± 0.6	2.50[e]

		2	0.26 ± 0.02	19 ± 2	11.7[e]
		3	0.28 ± 0.02	74 ± 6	43.7[e]
10	1.178	1	0.46 ± 0.04	7.4 ± 0.7	2.66[e]
		2	0.24 ± 0.04	16 ± 2	10.9[e]
		3	0.30 ± 0.02	77 ± 5	42.2[e]

[a] Chi-squared, a measure of goodness of fit. [b] Normalized pre-exponential factor for *i*th component. [c] Fractional contribution of the ith component. [d] Lifetime of the *i*th component. [e] The errors generated were not physically meaningful, so have not been quoted

3.4. Upconversion lifetimes: TTA-UC pair-doped ureasils

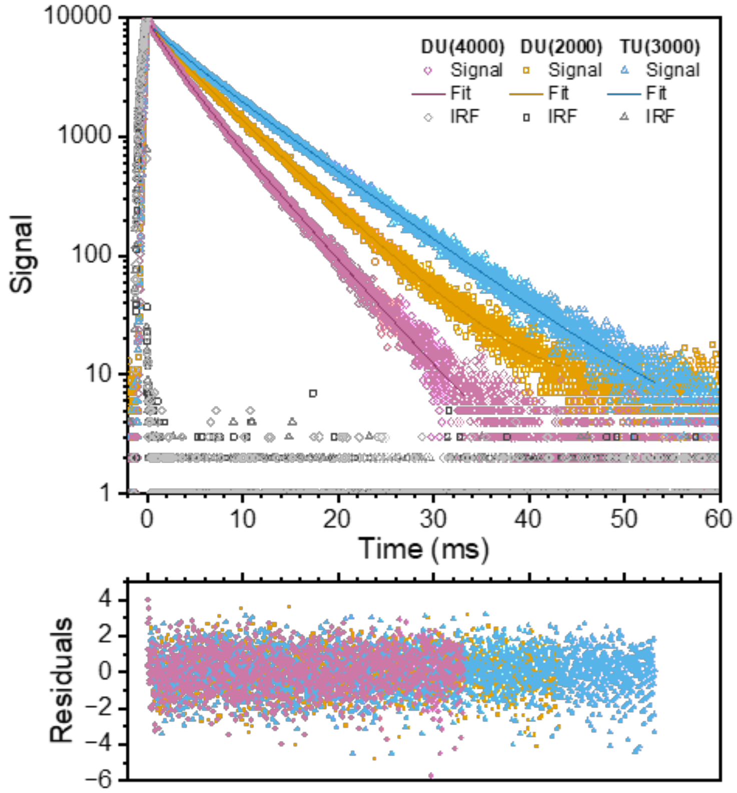

Figure S7. Upconversion decays and IRF (λ_{ex} = 532 nm, λ_{em} = 440 nm), decay fits (solid lines) corresponding residuals forf PdOEP (0.1 mM) and DPA (10 mM) in ureasil hosts, measured in a sealed cuvette after purging with N_2 for 4 hours.

Table S9. Multiexponential fitting parameters for upconversion decays (λ_{ex} = 532 nm, λ_{em} = 440 nm) of PdOEP (0.1 mM) and DPA (10 mM) in ureasil hosts, measured in a sealed cuvette after purging with N_2 for 4 hours.

Ureasil	χ^{2} [a]	*i*	α_i [b]	f_i (%)[c]	$\tau_{UC,i}$ (ms)[d]	<τ_{UC}> (ms)[e]
DU(4000)	1.538	1	0.40[f]	22.8 ± 0.4	2.2[f]	4.21 ± 0.05
		2	0.60[f]	77.2 ± 1.1	4.8[f]	
DU(2000)	1.390	1	0.21[f]	9.8 ± 0.4	2.5[f]	5.57 ± 0.06
		2	0.79[f]	90.2 ± 1.0	5.9[f]	
TU(3000)	1.400	1	0.21[f]	9.7 ± 0.3	3.0[f]	7.15 ± 0.06
		2	0.79[f]	90.3 ± 0.8	7.6[f]	

[a] Chi-squared, a measure of goodness of fit. [b] Normalized pre-exponential factor for *i*th component. [c] Fractional contribution of the ith component. [d] Lifetime of the *i*th component. [e] Average lifetime. [f] The errors generated were not physically meaningful, so have not been quoted.

4. TTET Efficiencies: TTA-UC pair-doped samples

The efficiency of triplet-triplet energy transfer (Φ_{TTET}) was calculated from the integrated phosphorescence decay intensities for PdOEP-doped samples (I_0) and TTA-UC pair-doped samples (I_{S+E}), both measured in a sealed cuvette after purging with N_2 for 4 hours:

$$\Phi_{TTET} = 1 - \frac{I_{S+E}}{I_0} \quad \text{(Eq.S13)}$$

Table S10. TTET efficiencies for PdOEP (0.1 mM) and DPA (10 mM) in ureasil hosts

**Ureasil**	I_0	I_{S+E}	Φ_{TTET} **(%)**
DU(4000)	1150 ± 30	56 ± 2	95.2 ± 0.2
DU(2000)	1300 ± 60	76 ± 5	94.2 ± 0.5
TU(3000)	1260 ± 140	74 ± 3	94.1 ± 07

5. References

(1) *Principles of Fluorescence Spectroscopy*; Lakowicz, J. R., Ed.; Springer US: Boston, MA, 2006. https://doi.org/10.1007/978-0-387-46312-4.

(2) Porrès, L.; Holland, A.; Pålsson, L.-O.; Monkman, A. P.; Kemp, C.; Beeby, A. Absolute Measurements of Photoluminescence Quantum Yields of Solutions Using an Integrating Sphere. *J Fluoresc* **2006**, *16* (2), 267–273. https://doi.org/10.1007/s10895-005-0054-8.

(3) Ahn, T.-S.; Al-Kaysi, R. O.; Müller, A. M.; Wentz, K. M.; Bardeen, C. J. Self-Absorption Correction for Solid-State Photoluminescence Quantum Yields Obtained from Integrating Sphere Measurements. *Review of Scientific Instruments* **2007**, *78* (8), 086105. https://doi.org/10.1063/1.2768926.

(4) de Mello, J. C.; Wittmann, H. F.; Friend, R. H. An Improved Experimental Determination of External Photoluminescence Quantum Efficiency. *Advanced Materials* **1997**, *9* (3), 230–232. https://doi.org/10.1002/adma.19970090308.

(5) Zhou, Y.; Castellano, F. N.; Schmidt, T. W.; Hanson, K. On the Quantum Yield of Photon Upconversion via Triplet–Triplet Annihilation. *ACS Energy Lett.* **2020**, *5* (7), 2322–2326. https://doi.org/10.1021/acsenergylett.0c01150.

(6) Douglas, P.; Eaton, K. Response Characteristics of Thin Film Oxygen Sensors, Pt and Pd Octaethylporphyrins in Polymer Films. *Sensors and Actuators B: Chemical* **2002**, *82* (2), 200–208. https://doi.org/10.1016/S0925-4005(01)01006-1.
